# The Ste20 kinase TAOK3 restrains Rac-driven cytoskeletal-mitochondrial coupling to preserve naive CD8^+^ T cell homeostasis and activation

**DOI:** 10.3389/fimmu.2026.1838764

**Published:** 2026-06-15

**Authors:** Sahine Lameire, Lana Vandersarren, Andrew Brown, Philippe Pouliot, Sophie Janssens, Stijn Vanhee, Hamida Hammad, Bart N. Lambrecht

**Affiliations:** 1 Laboratory of Immunoregulation and Mucosal Immunology, VIB Center for Inflammation Research, Ghent, Belgium; 2 Department of Internal Medicine and Pediatrics, Ghent University, Ghent, Belgium; 3 Laboratory for ER Stress and Inflammation, VIB Center for Inflammation Research, Ghent, Belgium; 4 Department of Head and Skin, Ghent University, Ghent, Belgium; 5 Department of Pulmonary Medicine, Erasmus University Medical Center Rotterdam, Rotterdam, Netherlands

**Keywords:** cytoskeleton, naive CD8+ T cells, naive T cell homeostasis, Rac (Rac GTPase), TCR - T cell receptor

## Abstract

Cytoskeletal remodelling is central to naive T cell fitness, organizing receptor-proximal signaling and mechanotransduction during TCR engagement. However, how cytoskeletal dynamics are coordinated with TCR signaling to preserve naive T cell fitness remains incompletely defined. Here, we identify the *Sterile 20*-family member *Thousand and One Kinase 3* (TAOK3) as a kinase-dependent regulator of naive CD8^+^ T cell maintenance that couples TCR signal integration to cytoskeletal control. Genetic deletion or kinase inactivation of TAOK3 resulted in a profound, cell-intrinsic loss of naive CD8^+^ T cells. Despite enhanced sensitivity to TCR ligation and enhanced downstream signaling, proliferating CD8^+^ T cells did not survive *in vitro* and anti-viral CD8^+^ T cell immunity was compromised *in vivo* in the absence of TAOK3. Unbiased phospho-proteomic analysis of *Taok3*-deficient mice revealed altered phosphorylation of the Rac regulators *Dedicator of Cytokinesis* DOCK8 and DOCK10, alongside actin-membrane scaffolding proteins. Consistent with this, *Taok3*-deficient naive CD8^+^ T cells exhibited elevated basal actin polymerisation, excessive reactive oxygen species accumulation, mitochondrial hyperpolarisation, and reduced spare respiratory capacity. Pharmacologic Rac inhibition normalised cytoskeletal dynamics, corrected the heightened TCR sensitivity, and preferentially restored mitochondrial membrane potential. Collectively, these findings identify TAOK3 as a coordinator of membrane-proximal organisation and cytoskeletal regulation that calibrates Rac-dependent signaling, thereby linking TCR signal integration to mitochondrial fitness and long-term maintenance of the naive CD8^+^ T cell pool.

## Introduction

Naive T cells are actively maintained in a state of homeostatic quiescence characterized by low biosynthetic activity, residence in G0, and strict control of activation thresholds (1–3). This poised state is sustained through the integration of tonic T cell receptor (TCR) signals derived from self-peptide-MHC interactions, survival cues provided by interleukin-7 (IL-7), and spatially restricted sphingosine-1-phosphate (S1P) signalling that governs recirculation through secondary lymphoid organs and optimal mitochondrial metabolism (2, 4–7). Together, these inputs preserve a self-renewing pool of quiescent naive T cells that is capable of mounting rapid responses upon antigen encounter while avoiding inappropriate activation (1). Maintenance of this equilibrium requires precise calibration. Insufficient tonic signalling compromises survival, whereas excessive or improperly integrated signals can drive premature activation, differentiation, or attrition of the naive pool (2). Disruption of this balance has been implicated in impaired immune responses, defective vaccine efficacy, and susceptibility to immune-mediated pathology. How naive T cells integrate persistent low-affinity signals while maintaining long-term fitness remains an important unresolved question.

The actin cytoskeleton plays a central role in this process. Beyond its established function in cell migration and immune synapse formation, cytoskeletal remodelling shapes the spatial and temporal organisation of receptor-proximal signalling and contributes to mechanotransduction during TCR engagement. Actin dynamics influence both the amplitude and persistence of signalling events and thereby contribute to setting the threshold for T cell activation (8–10). However, the molecular mechanisms that couple cytoskeletal organisation to sustained naive T cell survival remain incompletely defined.

Among the principal regulators of actin dynamics are the Rho family of small GTPases, including Rac and Cdc42. These molecular switches cycle between inactive GDP-bound and active GTP-bound states and coordinate actin polymerisation, cell polarity, and adhesion while engaging a broad network of downstream effectors (11). In T cells, Rac signalling has been implicated not only in cytoskeletal remodelling but also in regulation of TCR signal strength, cellular metabolism, and survival (12). Rac-dependent pathways intersect with mitochondrial function and redox control, in part through regulation of NADPH oxidase activity and mitochondrial ROS production, suggesting a close functional coupling between cytoskeletal signalling and metabolic fitness, required for T cell survival in homeostasis, and proper effector function upon stimulation (13, 14).

The Thousand-and-one (TAO) kinases constitute a family of Ste20-like serine/threonine kinases with emerging roles in signal organisation and cellular stress responses. Mammals express three paralogs, TAOK1, TAOK2, and TAOK3, yet their functions in immune cells remain incompletely understood (15, 16). Previous work from our laboratory has identified TAOK3 as a regulator of membrane trafficking and fate decisions in B cells, where it controls delivery of the metalloprotease ADAM10 to the cell surface and thereby enables Notch-dependent marginal zone B cell differentiation (17). In dendritic cells, TAOK3 has likewise been implicated in regulation of activation-associated signalling pathways, that contribute to DC development and function (18, 19). In T cells, TAOK3 has been proposed to function as a rheostat of activation threshold by modulating inhibitory signalling circuits, suggesting that it may operate at an early point of signal integration (20, 21). However, whether TAOK3 contributes more broadly to the coordination of cytoskeletal organisation, signal tuning, and metabolic fitness in naive T cells has not been addressed.

Here, we investigate the role of TAOK3 in naive T cell homeostasis. Using genetic deletion and kinase-dead models, we show that TAOK3 is required in a cell-intrinsic manner for the maintenance of the naive T cell compartment. Loss of TAOK3 lowers the threshold for early TCR signal initiation, yet compromises the ability of naive CD8^+^ T cells to survive or clonically expand *in vivo*. Through unbiased phosphoproteomics and pharmacological interventions, we identify dysregulated Rac-dependent cytoskeletal signalling, accompanied by redox imbalance and impaired mitochondrial fitness, as central features of TAOK3 deficiency leading to T cell loss. These findings position TAOK3 as an organisational kinase that coordinates membrane-proximal signalling with cytoskeletal and metabolic programs to preserve long-term naive CD8^+^ T cell fitness.

## Results

### TAOK3 kinase activity is required for naive CD8^+^ T cell homeostasis

**Figure 1:**
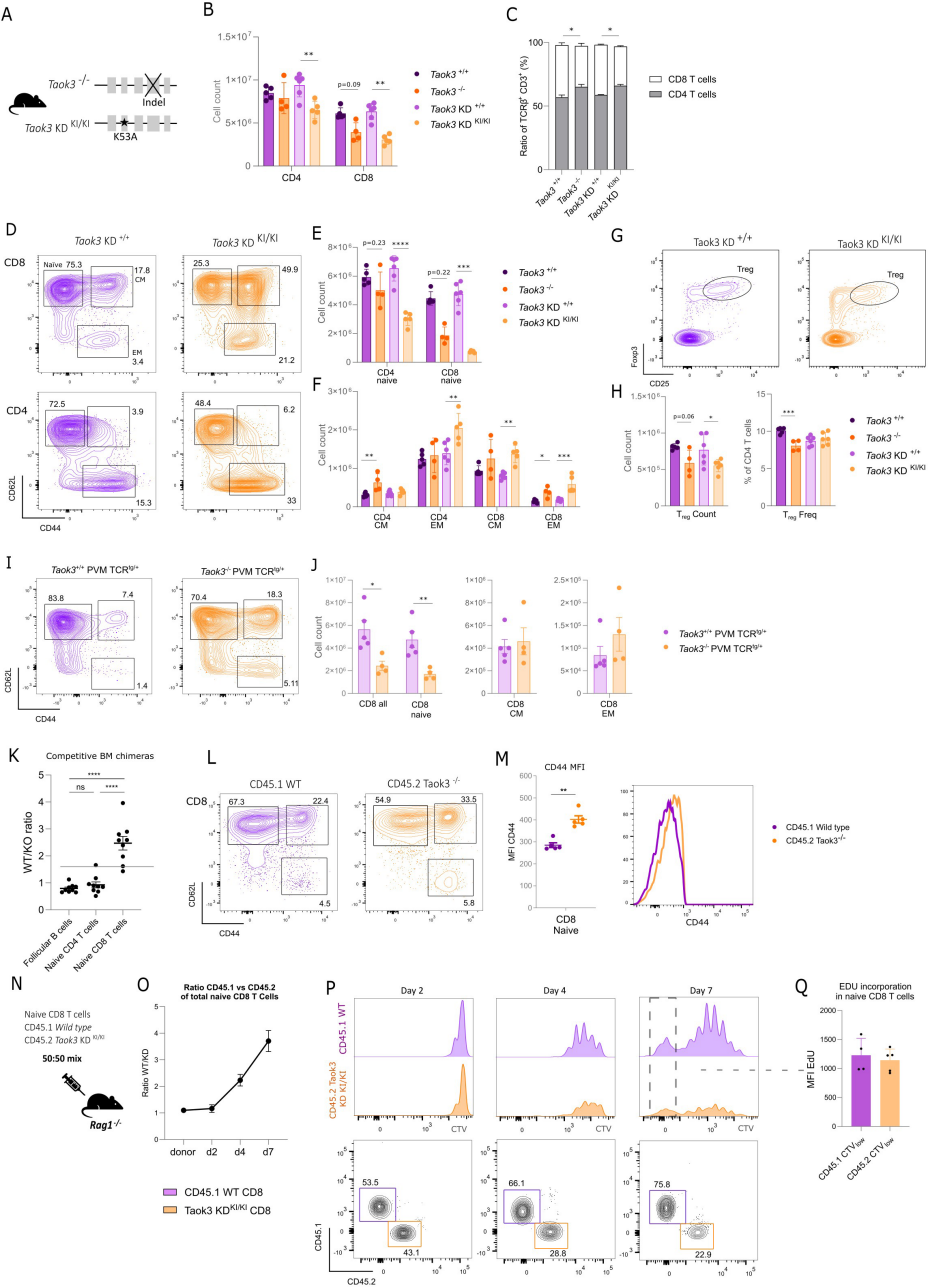
**(A)** Schematic representation of the genetic models used in this study. Taok3^-/-^ mice carry an exon deletion resulting in an indel, whereas Taok3 KD mice harbour a lysine-to-alanine substitution at position 53 (K53A) within the catalytic domain, abolishing TAOK3 kinase activity. **(B)** Absolute number of splenic CD4^+^ and CD8^+^ T cells. **(C)** Ratio of CD4^+^ to CD8^+^ T cells within the total splenic TCRβ^+^CD3^+^ T cell pool. **(D)** Representative flow cytometry plots of CD62L (L-selectin) vs CD44 showing naive, central memory (CM) and effector memory (EM) CD4^+^ and CD8^+^ splenic T cells. Cells were gated on viable CD45^+^CD11c^-^CD19^-^CD3^+^ lymphocytes, followed by CD4^+^ or CD8^+^ subsets. Numbers indicate percentages of the parent population. **(E)** Absolute numbers of naive CD4^+^ and CD8^+^ splenic T cells. **(F)** Absolute numbers of central memory (CM) and effector memory (EM) CD4^+^ and CD8^+^ splenic T cells. **(G)** Representative flow cytometry plots depicting Foxp3 and CD25 expression. Cells were gated on viable CD45^+^CD3^+^CD4^+^ T cells. **(H)** Absolute numbers and frequencies of splenic regulatory T cells (Foxp3^+^CD25^+^) within the CD4^+^ T-cell compartment. **(I)** Representative flow cytometry plots of CD62L (L-selectin) vs CD44 showing naive, central memory (CM) and effector memory (EM) CD8^+^ splenic T cells. Cells were gated on viable CD45^+^CD11c^-^CD19^-^CD3^+^CD8^+^ T cells. Numbers indicate percentages of the parent population. **(J)** Absolute numbers of total, naive, central memory (CM), and effector memory (EM) monoclonal CD8^+^ T cells. **(K)** Ratios of WT to KO cells in competitive bone marrow chimeras. Follicular B cells serve as an unaffected internal control **(L)** Representative flow cytometry plots of CD62L (L-selectin) vs CD44 showing naive, central memory (CM) and effector memory (EM) CD8^+^ splenic T cells, derived from CD45.1^+^ WT or CD45.2^+^ KO compartment in competitive bone marrow chimeras. Cells were gated on viable CD3^+^CD8^+^ lymphocytes. Numbers indicate percentages of the parent population. **(M)** CD44 mean fluorescence intensity (MFI) of naive CD8^+^ T cells derived from WT and KO donors in competitive bone marrow chimeras. Representative flow cytometry histograms are shown. Cells were gated on viable CD3^+^CD8^+^CD62L^+^CD44^low^. **(N)** Schematic representation of the competitive adoptive transfer experiment to assess homeostatic proliferation (n=3). **(O)** Ratio of CD45.1^+^ WT to CD45.2^+^ *Taok3* KD ^KI/KI^ donor-derived cells over time following competitive adoptive transfer. **(P)** Representative CTV dilution profiles and CD45.1 vs CD45.2 flow cytometry plots over time following competitive adoptive transfer. Numbers indicate percentages of the parent population. **(Q)** EdU incorporation at day 7 following competitive adoptive transfer, assessed within the proliferated donor-derived T-cell population. Statistical analysis Each dot represents an individual mouse. Data are representative of at least two independent experiments. **(B, E, F, H, J, K)** Normality was assessed by Shapiro–Wilk testing. Parametric or non-parametric tests were applied accordingly (one-way ANOVA with Bonferroni or Kruskal–Wallis with Dunn’s correction). **(C)**. Statistics were performed on CD4/CD8 ratios to account for compositionality (Kruskal–Wallis test), although data are displayed as percentages. **(M, Q)** Welch’s t-test. **(O)** One sample Wilcoxon signed-rank test. *P <.05, **P <.01, ***P <.001 and ****P<.0001.

Previously, we generated TAOK3 gene-deficient mice that showed defects in splenic marginal zone B cells and splenic DC subsets already in steady state (17, 19). To determine whether TAOK3 also regulates peripheral T cell homeostasis, we quantified CD4^+^ and CD8^+^ splenic T cell subsets in *Taok3*-deficient and littermate control *Taok3*^+/+^ wild type (WT) mice (**Figure 1A**). Loss of TAOK3 resulted in a reduction in peripheral CD8^+^ T cell numbers in adult mice, also evidenced by an increased CD4^+^/CD8^+^ ratio among total TCRβ^+^CD3^+^ lymphocytes compared with WT controls (**Figures 1B, C**). TAOK3 is a MAP kinase but potentially also functions as a scaffold protein recruiting other signalling intermediates (22). To discriminate these functions, we have previously generated kinase-dead (*Taok3* KD^KI/KI^) mice harbouring an 53Lys>Ala amino-acid substitution in the catalytic domain of TAOK3 (**Figure 1A**) (18). These mice essentially phenocopied not only the T cell alterations seen in *Taok3*^-/-^ mice, but also other previously described TAOK3-dependent phenotypes, supporting a critical role for its kinase activity (18, 23). In fact, the phenotype was slightly more pronounced, with reductions not only in CD8^+^ T cells, but also in splenic CD4^+^ T cell numbers compared with *Taok3* KD^+/+^ controls (**Figure 1B**). Based on the strong phenotypic overlap between both models, mouse lines are used interchangeably throughout the study, as indicated for each experiment.

In-depth phenotypic analysis revealed that the reduction of splenic CD4^+^ and CD8^+^ cells in *Taok3* KD^KI/KI^ mice was driven by loss of CD44^lo^CD62L^+^ naive T cells, accompanied by a reciprocal accumulation of CD44^hi^ CD62L^+^ T_CM_ recirculating central memory cells and CD44^hi^ CD62L^lo^ T_EM_ effector memory, indicative of an enhanced activation state in the absence of stimulation (**Figures 1D–F**). These effects were most marked in the CD8^+^ compartment of *Taok3* KD^KI/KI^ mutants, and were also seen in *Taok3*^-/-^ mice (**Figures 1E, F**). The number of CD4^+^Foxp3^+^CD25^+^ regulatory T cells were reduced in the absence of TAOK3 or kinase function. However, the relative frequency of Tregs among CD4^+^ T cells was preserved in *Taok3* KD^KI/KI^ mice but reduced in *Taok3*^^-^/^-^^ mice (**Figures 1G, H**).

No major alterations in TCRVβ usage were detected in peripheral CD8^+^ T cells or CD8^+^ single positive thymocytes (**Supplementary Figure S1A**), arguing against gross TCR repertoire skewing in *Taok3*^-/-^ mice. To exclude TCR repertoire-dependent contributions to the observed phenotype, we crossed *Taok3*^-/-^ mice to MHCI-restricted TCR transgenic mice expressing a receptor specific for the N_339–347_ peptide derived from the nucleoprotein of pneumonia virus of mice (PVM) (24). Also in this setting, there was a reduction in antigen-reactive CD8^+^ T cells in the absence of TAOK3 (**Figure 1J**). Consistent with results in mice with a polyclonal repertoire, the reduction in CD8 T cells in this TCR-transgenic setting was largely confined to the naive subset (**Figures 1I, J**).

Since *Taok3* deficient mice were mildly lymphopenic (17), it is possible that the transition of naive to memory phenotype in CD8^+^ T cells in these mice was driven by lymphopenia (25). To address this, and to further test whether the observed changes in CD8^+^ T cells were cell-intrinsic, we constructed competitive bone marrow chimera experiments, reconstituting irradiated CD45.1.2 recipient mice with a 50:50 mixture of CD45.1 WT and CD45.2 *Taok3*^-/-^ bone marrow cells. Eight weeks after reconstitution, the CD45.1/CD45.2 chimerism ratio for follicular B cells in the spleen was 0.8 ± 0.14, and a similar ratio was seen in CD4^+^ T cells (0.9 ± 0.31). In contrast, the chimerism ratio of CD8^+^ T cells was 2.5 ± 0.71, indicating that the defect in CD8^+^ T cells was cell-intrinsic (**Figure 1K**). Phenotypic analysis of donor-derived CD8^+^ T cells in the competitive setting further revealed that CD45.2^+^ Taok3^-/-^ cells exhibited a pronounced shift toward CD44^hi^ central and effector memory phenotypes compared with their CD45.1^+^ WT counterparts (**Figure 1L**). Notably, even naive *Taok3*^-/-^ donor-derived CD8^+^ cells displayed elevated surface CD44 expression, demonstrating that the loss of naive CD8^+^ T cells and their conversion toward a memory phenotype is cell-intrinsic and not secondary to lymphopenia (**Figure 1M**).

An important aspect of peripheral naive T cell homeostasis is the potential to respond to homeostatic cues for survival, including self-MHC interactions and homeostatic cytokines (2, 4–7). To address this, we performed competitive adoptive transfer experiments using FACS-sorted naive CD8^+^ T cells derived from CD45.1 WT or CD45.2 *Taok3* KD^KI/KI^ mutant mice into a *Rag1*^-/-^ lymphopenic hosts (**Figure 1N**), to force transferred cells into homeostatic proliferation (26). This approach revealed a progressive loss of *Taok3* KD^KI/KI^ naive CD8^+^ T cells relative to WT counterparts (**Figure 1O**). Two days after adoptive transfer however, equal numbers of CTV-labelled CD8^+^ lymphocytes were recovered from the spleen suggesting there were no immediate homing defects of injected cells. This was further supported by the fact that there were no differences in the CTV division profile at these early time points (**Figure 1O**). From day 4 onwards, however, the ratio of WT to *Taok3* KD^KI/KI^ CD8^+^ T cells progressively shifted in favour of wild type (**Figure 1O**), which could reflect altered proliferation, emigration or cell death of *Taok3* KD^KI/KI^ cells. Despite the fact that fewer *Taok3* KD^KI/KI^ cells were recovered, the CTV profile was indicative of comparable numbers of divisions, an effect that persisted up to 7 days after transfer (**Figure 1P**). In further support of intact proliferative capacity, the amount of EdU incorporation of cells as a measure of S-phase entry was identical between divided WT and *Taok3* KD^KI/KI^ mice (**Figure 1Q**). There were no signs of selective emigration of *Taok3* KD^KI/KI^ cells to the bloodstream, as similar phenotypes were consistently observed across spleen, lymph nodes, and peripheral blood. Together, these findings indicate that the reduced abundance of TAOK3-deficient naive CD8^+^ T cells reflects an intrinsic defect in competitive survival, rather than a failure to divide.

### TAOK3 sets the threshold for TCR responsiveness and is required for clonal expansion

**Figure 2:**
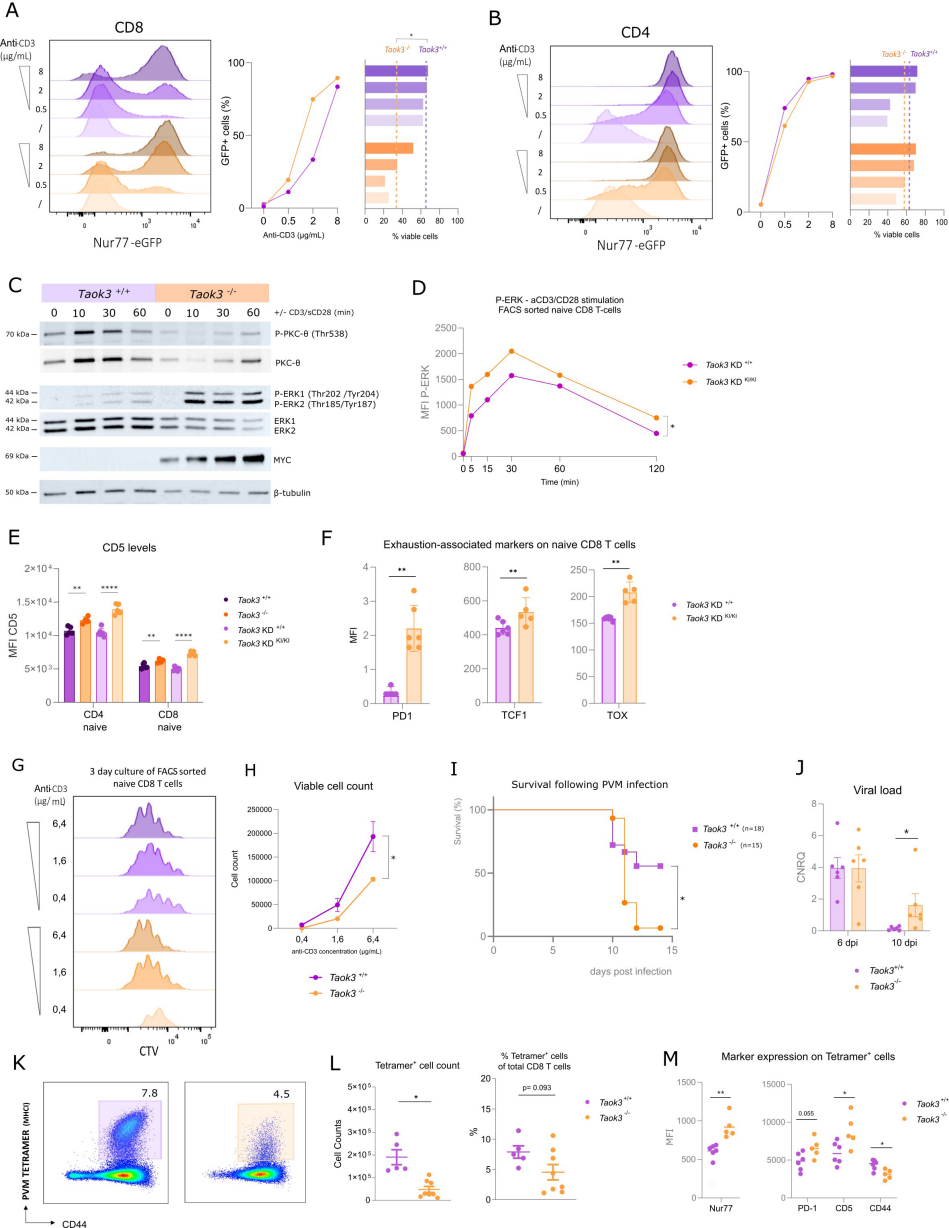
**(A)** Overnight culture of FACS-sorted naive CD8^+^ T cells stimulated with increasing concentrations of anti-CD3 in the presence of fixed anti-CD28 (2 µg/ml). Nur77-eGFP reporter activity was assessed by flow cytometry, with representative plots and quantification shown. The percentage of viable cells per condition is shown alongside. **(B)** Overnight culture of FACS-sorted naive CD4^+^ T cells stimulated with increasing concentrations of anti-CD3 in the presence of fixed anti-CD28 (2 µg/ml). Nur77-eGFP reporter activity was assessed by flow cytometry, with representative plots and quantification shown. The percentage of viable cells per condition is shown alongside. **(C)** Western blot analysis of signalling responses in isolated Taok3^+^/^+^ and Taok3^-^/^-^ naive CD8^+^ T cells at the indicated time points following activation with anti-CD3/CD28-coated beads (1:1 ratio T cell:bead). **(D)** ERK phosphorylation by phospho-flow cytometry in FACS-sorted naive T cells upon stimulation with plate-coated anti-CD3 (3 µg/mL) and soluble anti-CD28 (2 µg/mL). **(E)** CD5 mean fluorescence intensity (MFI) on naive splenic CD4^+^ and CD8^+^ T cells in TAOK3 deficient mice. **(F)** Expression of exhaustion-associated markers assessed by flow cytometry. Cells were gated on viable CD45^+^CD3^+^TCRβ^+^CD8^+^CD62L^+^CD44^low^ naive CD8^+^ T cells. **(G)** CTV dilution of FACS-sorted naive CD8^+^ T cells during multi-day culture with increasing anti-CD3 doses and fixed anti-CD28 (2 µg/mL). **(H)** Quantification of viability corresponding to panel G, showing FACS-sorted naive CD8^+^ T cells cultured for multiple days with increasing doses of anti-CD3 and a fixed concentration of anti-CD28 (2 µg/mL)(n=2). **(I)** Kaplan-Meier survival curve following intratracheal infection with LD_50_ dose of PVM strain J3666. Taok3^+/+^ (n=18) and Taok3^-/-^ mice (n=15) were monitored daily for survival throughout the course of infection. **(J)** Quantification of viral RNA levels quantified by qPCR at the indicated time points following intratracheal infection with PVM strain J3666. **(K)** Representative flow cytometry plots showing gating of N_339–347_ tetramer^+^ CD8^+^ T cells at 10 days post infection (dpi). **(L)** Absolute numbers (left) and frequencies (right) of N_339–347_ tetramer^+^ CD8^+^ T cells at 10 dpi, gated as shown in panel **(K, M)**. Marker expression on N_339–347_ tetramer^+^ CD8^+^ T cells at 10 dpi. Statistical analysis: **(A, B, D, H)**. Two-way ANOVA. **(E)** One-way ANOVA with Bonferroni correction. **(F, L, M)** Welch’s t-test. **(J)** Mann-Whitney test. **(I)** Log-rank test. **(E, F, J, L, M)** = each dot represents an individual mouse. **(A, B, D)** each data point represents an individual well. Data are representative of at least two independent experiments. *P <.05, **P <.01 and ****P<.0001.

Previous studies have suggested that TAOK3 may function as an activation rheostat in T lymphocytes, acting early during TCR signal integration, in part through modulation of SHP-1 activity (20, 21). To directly assess integrated TCR signalling, we crossed *Taok3^-^*^/-^ mice to the *Nr4a1*-GFP (Nur77-GFP) reporter mice, and measured Nur77 intensity as a quantitative readout of TCR signalling (27, 28). Stimulation with gradually increasing doses of anti-CD3 in the presence of fixed anti-CD28 led to enhanced Nur77-GFP induction in *Taok3*^-/-^ naive CD8^+^ T cells relative to WT controls, particularly at low levels of TCR engagement, while Nur77-GFP intensity converged at higher anti-CD3 doses (**Figure 2A**). Despite this heightened signalling under low-dose stimulation conditions, TAOK3-deficient naive CD8^+^ T cells failed to maintain survival *in vitro* (**Figure 2A**). These alterations were not observed in CD4^+^ T cells (**Figure 2B**), and were not due to TCR repertoire differences, since enhanced Nur77-GFP was also observed in PVM-TCR transgenic CD8^+^ T cells crossed to a *Taok3*^-/-^ background (**Supplementary Figure S2A**). Consistent with a lower activation threshold, we observed higher phosphorylation of ERK1 and ERK2, critical on-off checkpoints in naive CD8^+^ T cell activation when studied by western blotting (**Figure 2C**) and phospho-flow cytometry (**Figure 2D**) (29). Protein kinase theta (PKC-θ) is another early integrator of TCR signalling that has been linked to CD8^+^ T cell survival without affecting proliferation (30). The degree of PKC-θ phosphorylation at the kinase catalytic domain (Thr538 site) appeared unaltered when taking into account the total amount of PKC-θ between genotypes (**Figure 2C**). The duration and magnitude of induction of MYC, a critical transcription factor controlling metabolic activation and fitness of T cells, was considerably higher in *Taok3*^-/-^ compared with WT mice after stimulation with anti-CD3 and anti-CD28 (**Figure 2C**).

Near-threshold homeostatic self-MHC-TCR interactions continuously shape and maintain naive CD8^+^ T cells, and the history of this tonic signalling can be approximated by surface CD5 expression (31). In line with their heightened sensitivity to low-dose TCR stimulation *in vitro*, naive CD8^+^ T cells from Taok3^-/-^ mice exhibited elevated CD5 surface levels *in vivo*, indicative of altered tuning of tonic TCR signalling under homeostatic conditions (**Figure 2E**). Chronic TCR engagement is known to promote features of T cell exhaustion, and accordingly, naive CD8^+^ T cells of *Taok3*^-/-^ mice showed increased expression of the exhaustion-associated markers PD1, TCF1 and TOX (**Figure 2F**). We next examined how this altered signalling state translates into proliferative responses and survival of naive CD8^+^ T cells across a range of TCR stimulation intensity *in vitro*. Whereas the proliferation profiles of cells stimulated at higher antigenic doses was comparable between genotypes, *Taok3*-deficient cells showed impaired expansion at low to intermediate stimulation doses; in parallel, viability was consistently reduced across all conditions (**Figures 2G, H**).

To study the differentiation of naive T cells into functional effector cells, we subjected *Taok3*^+/+^ and *Taok3*^-/-^ mice to PVM infection, administered via the inhaled route, which serves as a murine orthologue model for the closely related RSV virus in humans. In this model, both viral clearance as well as lung immunopathology are known to strongly depend on CD8^+^ T cells (32, 33). Mice were infected with a sublethal dose (LD_50_) of PVM strain J3666 and survival was monitored over the course of infection. Survival analysis revealed a striking difference: 93% of *Taok3*^-/-^ mice succumbed to infection by day 12 post-infection (dpi), compared with 44% mortality in WT controls (**Figure 2I**). Correspondingly, viral load measurements at 10 dpi, when viral clearance in this model is usually achieved, showed significantly higher viral titers in *Taok3*^-/-^ lungs, suggesting impaired and delayed virus elimination (**Figure 2J**). Given the dual role of T cells in both antiviral defence and immunopathology in the PVM model (33), we next evaluated the magnitude of the virus-specific CD8^+^ T cell response. In this model, a marked T cell infiltration into the lungs is typically seen around 10 dpi (34). To quantify antigen-specific responses, we employed MHC class I tetramers loaded with the immunodominant N_339–347_ peptide (35) (**Figure 2K**). In *Taok3*^+/+^ mice, up to 2-4x10^5^ N_339-347_ -specific CD8^+^ T cells had infiltrated the lung by 10 dpi at the peak of disease severity (34) (**Figure 2L**). However, in *Taok3*^-/-^ mice 4 to 9-fold lower numbers of N_339-347_ -specific CD8^+^ T cells were detected in the lung, constituting only 0,5x10^5^ tetramer-positive CD8^+^ T cells on average. Data from a repeat experiment using Nur77-GFP reporter mice revealed elevated expression of CD5, PD-1, and Nur77 in the remaining tetramer-responsive *Taok3*^-/-^ CD8^+^ T cells, all markers associated with heightened TCR sensitivity, while induction of CD44 was reduced (**Figure 2M**).

These data indicate that loss of TAOK3 uncouples early TCR signal initiation from sustained signal integration required for clonal expansion and survival.

### TAOK3 constrains Rac-dependent cytoskeletal signalling and preserves redox-mitochondrial fitness

**Figure 3:**
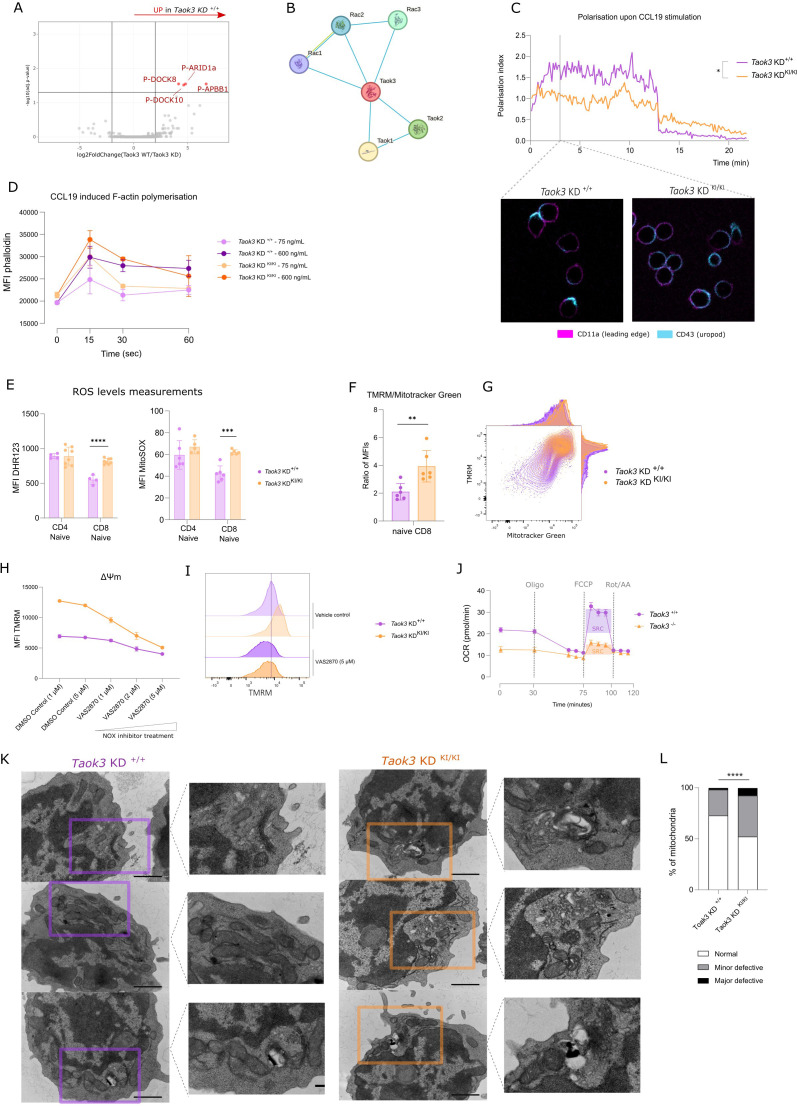
**(A)** Volcano plot of phosphoproteomic profiling of FACS-sorted naive CD8^+^ T cells, showing differential phosphosite abundance between experimental groups. Log_2_ fold change is plotted on the x-axis, significantly regulated phosphosites are highlighted in red (n=3). **(B)** STRING functional association network of TAOK3, generated using STRING v12 for *Mus musculus*. **(C)** Polarization index of FACS-sorted naive CD8^+^ T cells following CCL19 stimulation over time, normalised to baseline (mean of 9 wells derived from 3 biological replicates, each measured in technical triplate). Cell polarization was assessed by immunostaining for CD11a (leading edge) and CD43 (uropod). A representative snapshot at 3 min post-stimulation is shown. **(D)** F-actin content assessed by Acti-stain Phalloidin staining in naive CD8^+^ T cells. Splenocytes were stimulated with CCL19 over time and gated on viable CD45^+^CD3^+^TCRβ^+^CD8^+^CD62L^+^CD44^low^ cells (n=2). **(E)** Intracellular ROS levels in naive CD4^+^ and CD8^+^ T cells were quantified by DHR123 and MitoSOX staining using flow cytometry. Cells were gated on CD45^+^CD3^+^TCRβ^+^CD62L^+^CD44^low^ cells. **(F)** Mitochondrial polarization of naive CD8^+^ T cells measured by flow cytometry as the ratio of TMRM to MitoTracker Green staining. **(G)** Representative flow cytometry plots of TMRM and MitoTracker Green corresponding to panel **(F, H)**. Mitochondrial membrane polarisation in FACS-sorted naive CD8^+^ T cells following 1,5h *ex vivo* treatment with multiple doses of NADPH oxidase inhibitor VAS2870. Mitochondrial polarization was assessed by TMRM staining using flow cytometry (n=2). **(I)** Representative flow cytometry histograms of TMRM fluorescence intensity corresponding to panel **(H, J)**. Seahorse extracellular flux analysis of oxygen consumption rate (OCR) in purified naive CD8^+^ T cells. OCR was measured under basal conditions and following sequential treatment with oligomycin (Oligo), FCCP, and rotenone plus antimycin A (Rot/AA), as indicated. Graphs represent the mean ± SEM of four biological replicates, with each data point being calculated from averaged triplicate (TN) or quadruplet (TM) readings across 5 sequential time points right before oligo addition (basal OCR), or 3 sequential time points following oligo or FCCP treatment (maximal OCR)(n=4) **(K)** TEM analysis of naive CD8^+^ T-cell ultrastructure with representative high-magnification views of the Golgi apparatus, mitochondria, and autolysosomal compartments. Taok3-deficient cells display Golgi swelling, altered mitochondrial morphology, and increased autophagolysosomal structures. **(L)** Mitochondrial morphology was scored in a blinded manner and classified as normal, mildly defective, or severely defective based on shape, outer membrane integrity, cristae organisation, and evidence of mitophagy. 207 *Taok3 KD*^+/+^; 334 *Taok3 KD* ^KI/KI^ mitochondria, derived from *Taok3 KD*^+/+^ and 72 *Taok3 KD* ^KI/KI^ images of randomly selected cells. Statistical analysis: **(C)** Two-way ANOVA. **(E, F)** Welch’s T test. **(L)** χ² test of independence. Data are representative of at least two independent experiments. E,F = each dot represents an individual mouse. For all other panels, this is indicated in the corresponding figure legends. *P <.05, **P <.01, ***P <.001 and ****P<.0001.

To identify molecular pathways altered by loss of TAOK3, and informed by the dependence of the observed phenotype on TAOK3’s kinase activity, we performed an unbiased quantitative proteomic and phosphoproteomic profiling of naive CD8^+^ T cells from *Taok3* KD^+/+^ and *Taok3* KD ^KI/KI^ mice. Differential abundance analysis revealed increased expression of Annexin A2 (ANXA2) and N-myc downstream regulated gene 1 (NDRG1), respectively previously implicated in plasma membrane dynamics and stress responses (36, 37) (**Supplementary Figure S3**). Phosphoproteomic analysis identified reduced phosphorylation of only a limited set of proteins, but notably the Rac/Cdc42 guanine nucleotide exchange factors *Dedicator of Cytokinesis* DOCK8 and DOCK10, as well as ARID1A and APBB1ip (**Figure 3A**). Given the established roles of DOCK proteins in actin remodelling and immune synapse formation in T cells, these findings suggested that TAOK3 may regulate Rho GTPase signalling. Moreover, TAOK3 has previously been picked up in a large interactome study as an interactor of the Rac family GTPases, as captured in the STRING interaction network (**Figure 3B**) (38).

To explore whether TAOK3 deficiency broadly affects cytoskeletal behaviour, we initially probed for Rac-dependent cytoskeletal remodelling in T cells as an exploratory approach. We examined CCR7-driven polarization of naive CD8^+^ T cells in response to CCL19, the chemokine involved in recruiting naive T cells to lymph nodes. Cell polarity was assessed by CD11a and CD43 redistribution as markers of leading-edge and uropod formation, respectively, serving as an indirect readout of underlying actin dynamics (39, 40). Consistent with a defect in cytoskeletal control, *Taok3*-deficient naive CD8^+^ T cells failed to establish stable front-rear polarity in response to CCL19 stimulation, as assessed by polarization index measurements (**Figure 3C**). In a parallel approach, flow cytometric analysis revealed slightly elevated basal polymerised F-actin levels in *Taok3* KD ^KI/KI^ cells, suggestive of an overactive upstream actin-regulatory program (**Figure 3D**). Upon CCL19 stimulation, these cells displayed exaggerated actin polymerisation across low and high chemokine ligand concentrations, suggesting that this phenotype results from disinhibited actin signalling rather than differences in receptor occupancy, consistent with altered regulation of actin dynamics (**Figure 3D**).

Because Rac signalling interfaces with redox regulation and mitochondrial function (41, 42), and both are closely linked to cell survival, we next assessed ROS levels and mitochondrial parameters. *Taok3*-deficient naive CD8^+^ T cells exhibited increased cellular and mitochondrial ROS production, as measured by increased staining with the DHR123 and MitoSOX probes, respectively (**Figure 3E**), whereas such changes were not apparent in naive CD4^+^ T cells. At the same time, *Taok3*-deficient naive CD8^+^ T cells display an increased mitochondrial membrane potential (Δψm) relative to mitochondrial mass, as assessed by elevated tetramethylrhodamine methyl ester (TMRM)-to-MitoTracker Green ratios, indicative of mitochondrial hyperpolarisation (**Figures 3F, G**). The mitochondrial hyperpolarisation was consistent with excessive NADPH oxidase (NOX) activity, a complex in which Rac GTPases serve as essential regulatory subunits, since the use of the pan-NOX inhibitor VAS2870 restored TMRM staining to the level of *Taok3* KD^+/+^ T cells (**Figures 3H, I**).

To further analyse the mitochondrial fitness and respiration of T cells, a Seahorse experiment was performed on equal amounts of sorted naive CD8^+^ T cells from *Taok3*^+/+^ and *Taok3*^-/-^ mice, and oxygen consumption rate (OCR) measured. In these experiments, *Taok3*-deficient cells demonstrated lower basal OCR, and were much less able to increase OCR upon electron chain uncoupling using FCCP compared with *Taok3* sufficient CD8^+^ T cells, resulting in a marked reduction in spare respiratory capacity (SRC), a key indicator of mitochondrial fitness in CD8^+^ T cells (43, 44) (**Figure 3J**). Investigator blinded ultrastructural analysis confirmed mitochondrial abnormalities and increased autophagolysosomal structures consistent with cellular stress (45) (**Figures 3K, L**).

Together, these data indicate that loss of TAOK3 results in disinhibited Rac-dependent cytoskeletal signalling coupled to redox imbalance, and impaired mitochondrial fitness, despite mitochondrial hyperpolarisation in naive CD8^+^ T cells.

### Rac inhibition rescues cytoskeletal and TCR signalling in TAOK3-deficient naive CD8^+^ T cells

**Figure 4:**
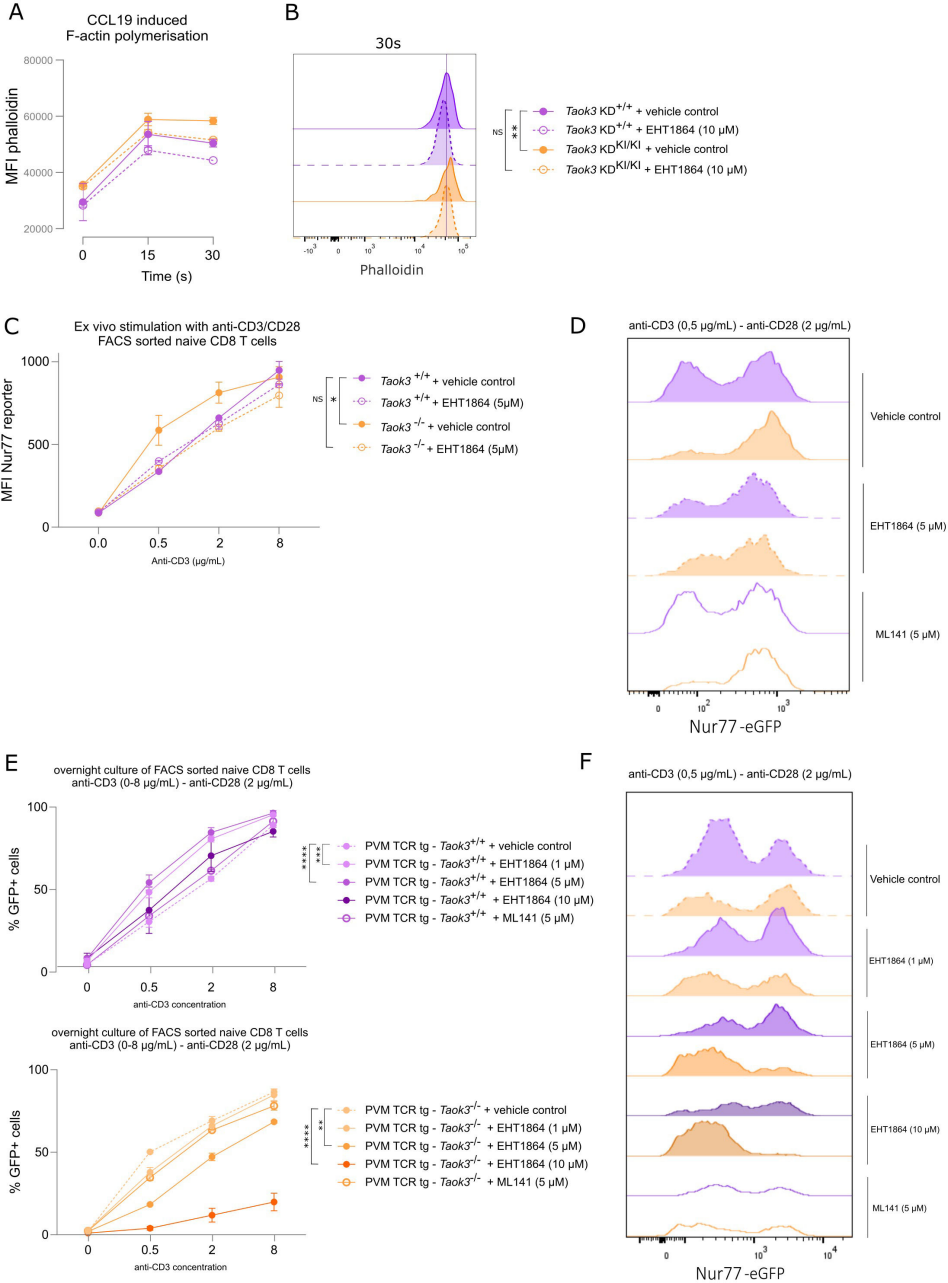
**(A)** Actin polymerisation in naive CD8^+^ T cells following CCL19 (400 ng/mL) stimulation. Splenocytes were stimulated with CCL19 over time. F-actin content was assessed by phalloidin staining using flow cytometry. Cells were treated with EHT1864 (10 µM) or matching vehicle control. Cells were gated via viable CD45^+^CD3^+^TCRβ^+^CD8^+^CD62L^+^CD44^low^. **(B)** Representative flow cytometry histograms of Phalloidin fluorescence intensity corresponding to panel A, shown for the condition after 30 sec of stimulation. **(C)** Overnight culture of FACS-sorted naive CD8^+^ T cells stimulated with increasing doses of anti-CD3 in the presence of fixed anti-CD28 (2 µg/mL). Nur77-eGFP reporter activity was assessed by flow cytometry. **(D)** Representative flow cytometry histograms corresponding to panel C, shown for cells stimulated with 0.5 µg/mL anti-CD3. **(E)** Overnight culture of FACS-sorted monoclonal naive CD8^+^ T stimulated with increasing doses of anti-CD3 in the presence of fixed anti-CD28 (2 µg/mL). Nur77-eGFP reporter activity was assessed by flow cytometry. **(F)** Representative flow cytometry histograms corresponding to panel E, shown for cells stimulated with 0.5 µg/mL anti-CD3. Statistical analysis: **(A, C)** Two-way ANOVA with Šídák’s multiple comparisons test. **(E)** Two-way ANOVA with Dunnett’s multiple comparisons test. Each dot represents the mean of three biological replicates. Data are representative of at least two independent experiments. *P <.05, **P <.01, ***P <.001 and ****P<.0001.

To determine whether Rac hyperactivity was causally related to the observed CD8^+^ T cell phenotypes of *Taok3*-deficient and kinase dead mice, we inhibited Rac function using a pharmacological inhibitor. EHT1864 blocks Rac by directly binding the Rac protein, inducing nucleotide release, and locking it in an inactive conformation that cannot activate downstream effectors. Rac inhibition indeed dampened exaggerated actin polymerisation responses, restoring F-actin dynamics toward levels seen in WT CD8^+^ cells (**Figures 4A, B**). In contrast, the inhibitor ML141 that more specifically targets Cdc42 did not reproduce these effects (**Supplementary Figure S4**).

We next assessed whether Rac hyperactivation underlies the increased responsiveness of *Taok3*^-/-^ CD8^+^ T cells to low level TCR stimulation in the presence of CD28 co-stimulation. Under low-dose anti-CD3 stimulation, Rac inhibition using EHT1864 selectively normalised Nur77-GFP induction in *Taok3*-deficient naive CD8^+^ T cells, whereas the vehicle control and Cdc42 inhibition had no effect (**Figures 4C, D**). Because TCR affinity can influence the dependence of lymphocyte activation on Rac, and to control for potential differences in repertoire selection in *Taok3*^-/-^ mice, we also performed inhibition experiments in a monoclonal PVM-TCR transgenic setting. Low-dose Rac inhibition modestly increased activation in *Taok3*^+/+^ CD8^+^ T cells whereas higher doses had minimal impact on their activation threshold. In contrast, low-level Rac inhibition normalised the exaggerated activation response of *Taok3*-deficient cells to levels seen in *Taok3*^+/+^ cells, while higher doses almost completely suppressed activation in the deficient cells (**Figures 4E, F**). These dose-dependent effects indicate that *Taok3*-deficient T cells have become functionally dependent on elevated Rac signalling, rendering them hypersensitive to Rac inhibition.

## Discussion

The spatial organisation of membrane receptors and signalling complexes depends on cytoskeletal scaffolding. Perturbations in cytoskeletal control are therefore expected to directly affect TCR signalling thresholds and cell survival. Upstream of actin dynamics, extracellular cues are integrated through Rho family GTPases, including Rac1, Cdc42, and RhoA, which coordinate cytoskeletal organisation and signalling competence. Consistent with this framework, impaired naive T cell survival occurs in mice lacking core components of the Rac signalling axis such as Vav1, WASP, and Nck (46–49), underscoring the importance of Rac-centered networks in T cell homeostasis.

Phosphoproteomic profiling of naive CD8^+^ T cells revealed altered phosphorylation of the Rac/Cdc42 guanine nucleotide exchange factors DOCK8 and DOCK10, implicating dysregulated Rho GTPase control in *Taok3*-deficient mice. DOCK proteins mediate spatially restricted Rac activation to ensure localized actin remodelling (50). Disruption of this control would be predicted to result in excessive or ectopic Rac activity, consistent with the elevated basal and induced F-actin observed in TAOK3-deficient naive CD8^+^ T cells. Altered Annexin A2 expression (36), identification of DOCK10 in *Taok3* overexpressing assays (51), prior evidence of TAOK3-Rac interaction (38), and the demonstration that the Ste20 kinase Mst1 regulates DOCK8-dependent Rac activation (52), together support a model in which TAOK3 restrains DOCK-mediated Rac signalling. Moreover, the loss of naive CD8^+^ T cells reported here, along with previously described defects in marginal zone B cells (17) and cDC2 migration (18), closely resemble phenotypes observed in DOCK8 deficiency (48, 53, 54), suggesting functional convergence between TAOK3 and DOCK-dependent cytoskeletal pathways.

Rac signalling also interfaces with NADPH oxidase-dependent ROS production and mitochondrial regulation. TAOK3-deficient naive CD8^+^ T cells exhibited elevated cellular and mitochondrial ROS, increased mitochondrial membrane potential relative to mitochondrial mass, and ultrastructural evidence of stress. Despite hyperpolarisation, these cells showed reduced basal oxygen consumption and markedly impaired FCCP-induced respiration, resulting in a substantial loss of spare respiratory capacity (SRC), a key indicator of mitochondrial fitness (43, 55). The combination of elevated Δψm and reduced SRC indicates dysregulated mitochondrial homeostasis rather than enhanced oxidative capacity (43). TAOK3 therefore appears to preserve mitochondrial adaptability by limiting basal Rac-dependent redox tone.

A defining feature of TAOK3 deficiency is the dissociation between early TCR signal initiation and sustained cellular fitness. TAOK3-deficient naive CD8^+^ T cells displayed enhanced Nur77 induction, increased ERK phosphorylation, and elevated MYC expression under low-level stimulation, consistent with a lowered activation threshold. However, they failed to maintain viability and competitive persistence, and showed evidence of heightened tonic sensing and exhaustion *in vivo*. Because naive CD8^+^ T cells rely on continuous low-level self–MHC class I signalling for survival (3, 56, 57), effective integration of these tonic cues requires coordinated cytoskeletal and mitochondrial adaptation. In TAOK3-deficient cells, exaggerated proximal signalling occurs in a metabolically constrained context where reduced SRC limits the ability to buffer energetic stress. This uncoupling likely drives attrition of the naive CD8^+^ pool and emergence of an exhausted phenotype.

The physiological impact of this defect is evident during viral infection. TAOK3-deficient mice exhibited increased mortality, delayed viral clearance, and markedly reduced accumulation of antigen-specific CD8^+^ T cells in the lung. Although residual virus-specific cells displayed heightened TCR sensitivity, clonal expansion and effector accumulation were severely compromised. Protective antiviral CD8^+^ T cell responses require rapid proliferation and robust metabolic reprogramming, with effector and memory cells relying on mitochondrial flexibility and SRC to meet fluctuating energetic demands (44). The reduction in SRC observed in TAOK3-deficient naive CD8^+^ T cells likely limits their ability to generate energetically competent effectors. Thus, impaired antiviral immunity in the absence of TAOK3 may reflect failure to sustain metabolic demands rather than defective signal initiation.

TAOK family kinases are established regulators of cytoskeletal organisation. TAOK1 and TAOK2 control microtubule and actin dynamics (58–63), whereas TAOK3 has been only indirectly linked to Rac1/3 signalling and stress responses (38, 64). Our findings extend the cytoskeletal role of the TAOK family to TAOK3 and demonstrate that it helps restrain Rac-dependent actin dynamics and associated redox pathways, thereby shaping proximal TCR signalling. Recent work implicates TAOK3 in regulating TCR thresholds via SHP-1 stability and Lck phosphorylation (20, 21). These mechanisms likely operate in parallel: proximal TCR signalling, SHP-1-Lck regulation, Rac-driven cytoskeletal remodelling, and metabolic adaptation are tightly coupled processes. TAOK3 appears to function as a nodal kinase aligning signal strength with structural and metabolic capacity, however, the precise causal relationships between these processes remain to be fully elucidated.

Collectively, our data support a model in which TAOK3 acts as a cytoskeletal–metabolic rheostat in naive CD8^+^ T cells. By constraining basal Rac-dependent actin dynamics and limiting NOX-driven redox tone, TAOK3 preserves mitochondrial respiratory reserve and calibrates TCR signal integration. In its absence, exaggerated Rac signalling elevates mitochondrial polarisation and ROS accumulation, reducing spare respiratory capacity and distorting activation thresholds. MYC-driven anabolic programs may proceed despite compromised mitochondrial resilience, permitting initial proliferation but undermining long-term survival. TAOK3 thus enforces alignment between cytoskeletal organisation, redox balance, and mitochondrial fitness, safeguarding naive T cell homeostasis and effective antiviral CD8^+^ immunity. Disruption of this coordination uncouples signal initiation from cellular endurance, ultimately compromising immune resilience.

## Materials and methods

### Mice

Whole body knock-out *Taok3*^-/-^ was generated by the Transgenic Core Facility (TCF) of the Inflammation Research Center (IRC) of VIB-UGent by injecting C57BL/6J zygotes with RNP complexes with gRNAs targeting a region in intron 5 (5′GGGTAACTGTGGTGACTTTG 3′) and a region in intron 6 (5′GGAGGCTGAGGCGGAACCAA 3′), resulting in the deletion of exon 6 (19). TAOK3 kinase-dead (KD) mice (*Taok3* KD, C57BL/6J-Taok3<em5Irc>/Irc MGI:8330839 were generated in house by the TCF of the IRC with the Easi-CRISPR method (65) by injecting along single-stranded DNA repair template containing exon 4 with an AAG-to-GCC mutation, causing a lysine-to-alanine mutation in position 53 of the Taok3 protein, predicted to be the catalytic site of Taok3 kinase domain (18). Nur77-eGFP reporter mice (RRID: IMSR_JAX:016617) were purchased at The Jackson Laboratory (USA) and have the *Nr4a1* (Nur77) promoter driving expression of a green fluorescent protein fusion protein. The generation of MHC class I-restricted PVM N339–347 PVM-specific CD8^+^ TCR transgenic mice is described in full detail in (24).

#### PVM infection

Mouse-passaged stocks of PVM strain J3666 were grown as described (66). Mice were anesthetized with isoflurane (2 l/min, 2-3%) and then infected intratracheally with a previously *in vivo* titrated sub-lethal dose of PVM in 80 µl PBS. Animals were monitored daily for body weight loss and clinical signs of disease, and humane endpoints were applied in accordance with predefined criteria. All experimental procedures were in accordance with institutional guidelines for animal care of the VIB site Ghent - Ghent University, and were approved under accreditation n° EC2021-091.

#### Generation of BM chimeras

Mixed bone marrow chimeric mice were generated by lethal irradiation (10 Gy) of CD45.1.2 C57BL/6 acceptor mice and subsequent intravenous injection of 2.10^6^ cells from a 50:50 mix of CD45.1 WT and CD45.2 Taok3^−/−^ bone marrow in a total volume of 100 µl. Eight to ten weeks after reconstitution, the immune organs of chimeric mice were analysed by flow cytometry for the ratios of CD45.1 WT to CD45.2 Taok3^-/-^ cells in different immune cell populations.

### Tissue sampling and processing for flow cytometry

Mice were sacrificed by cervical dislocation or CO_2_ asphyxiation. Thymus, spleen, and lymph nodes were harvested and mechanically dissociated through 70-µm cell strainers (Corning, #352350) to obtain single-cell suspensions. Cells were incubated with fluorescently labelled monoclonal antibodies (see **Table 1**) for 30 min at 4 °C in the presence of anti-mouse FcγRII/III (clone 2.4G2) to reduce nonspecific binding. Dead cells were excluded using Fixable Viability Dye eFluor™ 780 (Thermo Fisher Scientific, #65-0865-14).

**TABLE 1:** Antibodies used for flow cytometry.

Antibody name	Identifier	Source
CD44	MCA1014FT	Bio-Rad
CD44	TONB80-0441-U100	Tonbo Biosciences
CD62L	104436	BioLegend
CD62L	104408	BioLegend
TCR beta	109206	BioLegend
TCR beta	109234	BioLegend
Donkey anti-Rabbit IgG	A-31573	Thermo Fisher Scientific
CD8a	100722	BioLegend
CD8b	747505	BD Biosciences
CD4	563790	BD Biosciences
CD4	100548	BioLegend
CD3e	100312	BioLegend
CD3e	612771	BD Biosciences
CD11c	61-0114-82	Thermo Fisher Scientific
CD5	740842	BD Biosciences
CD45	568336	BD Biosciences
Qa2	558973	BD Biosciences
Streptavidin	405225	BioLegend
CD24	15-0242-82	Thermo Fisher Scientific
CD25	45-0251-82	Thermo Fisher Scientific
CD25	102036	BioLegend
Foxp3	17-5773-82	Thermo Fisher Scientific
CD45.1	553776	BD Biosciences
CD45.2	612778	BD Biosciences
CD279	109110	BioLegend
Ly108	748564	BD Biosciences
TOX	12-6502-82	Thermo Fisher Scientific
HIF-1α	52496	Cell Signaling Technology
TIM-3	119725	BioLegend
TCF1	6444S	Cell Signaling Technology
CD69	566500	BD Biosciences
c-kit	17-1171-83	Thermo Fisher Scientific
MHC-II	12-5321-83	Thermo Fisher Scientific
CD11c	553802	BD Biosciences
Ly-6G	551461	BD Biosciences
CD11b	553311	BD Biosciences
TCR gamma/delta	15-5711-81	Thermo Fisher Scientific
CD19	25-0193-82	Thermo Fisher Scientific
F4/80	123114	BioLegend
Ter119	25-5921-82	Thermo Fisher Scientific
Phospho-ERK1/2 Thr202/Tyr204)	369506	Biolegend
PVM MHCI (H2-Db, N339) (GAPRNRELF)	Custom made	BioLegend

For proliferation assays, cells were labelled with Cell Proliferation Dye eFluor™ 450 (CTV; Thermo Fisher Scientific) according to the manufacturer’s instructions. Absolute cell numbers were determined by adding 10 µL/sample of e123count eBeads (Thermo Fisher Scientific, #01-1234-42).

For intracellular staining of cytosolic proteins or actin, cells were fixed and permeabilized using BD Cytofix/Cytoperm™ (BD Biosciences, #554714). For nuclear antigen detection, cells were fixed and permeabilized after surface staining using the eBioscience™ Foxp3/Transcription Factor Staining Buffer Set (Thermo Fisher Scientific, #00-5523-00).

Phosphorylation status was assessed using a BD Phosflow protocol. At the end of stimulation, cells were fixed immediately by adding an equal volume of pre-warmed (37 °C) BD Cytofix™ Fixation Buffer (BD Biosciences, #554655; equivalent to Phosflow Fix Buffer I) and incubated for 10 min at 37 °C. Cells were washed with FACS buffer (PBS supplemented with BSA and EDTA) and centrifuged (400 × g, 5 min, 4 °C). Surface staining was performed for 20 min on ice in the dark, followed by permeabilization with ice-cold (−20 °C) BD Phosflow™ Perm Buffer III (BD Biosciences, #558050) for 30 min on ice. Samples were washed and rehydrated in cold FACS buffer. Intracellular staining was performed sequentially with 45-min incubations at 4 °C, with washes between steps. Samples were acquired on the same day. All samples included fluorescence minus one (FMO) and single-stain controls.

Mitochondrial membrane potential was assessed using TMRM (Thermo Fisher Scientific, #I34361) according to the manufacturer’s instructions (non-wash protocol; cells maintained at 37 °C during acquisition). Mitochondrial mass was evaluated using MitoTracker™ Green FM (Thermo Fisher Scientific, #M46750). Reactive oxygen species were measured using DHR123 (Cayman Chemical, #85100) and MitoSOX™ (M36005, Thermo Fisher Scientific). F-actin was detected using Acti-stain Fluorescent Phalloidin 488 (Tebu-bio, #PHDG1-A). TCR Vβ repertoire analysis was performed using the TCR Vβ Screening Panel kit (BD Biosciences).

Data were acquired on a BD FACSymphony™ A5, BD FACSymphony™ A3, or BD LSRFortessa™ and cells were sorted using a BD FACSAria™ II or III (all BD Biosciences). Photomultiplier tube voltages were optimized prior to acquisition. Compensation matrices were calculated using single-stained UltraComp eBeads (Thermo Fisher Scientific). Data were analyzed using FlowJo (Tree Star) and GraphPad Prism 10 (GraphPad Software).

#### Adoptive transfer

Spleens were collected from CD45.1 WT and CD45.2 Taok3 KD^KI/KI^ and were dispersed through a 70μm cell strainer. Naive CD8^+^ were sorted on BD ARIA FACS based on viability and CD45^+^CD11c^-^CD19^-^CD8^+^CD62L^+^ CD44^low^. Purified naive CD8^+^ T cells were mixed in a 50:50 ratio and a total of 1.10^6^ naive CD8^+^ T cells in 100µl endotoxin-low PBS were adoptively transferred by i.v injection in the lateral tail vein of CD45.1.2 recipients. The CD45.1:CD45.2 ratio of the mixed CD8^+^ T-cell suspension was determined at day 0 (input) and reassessed at days 2,4 and 7 post-transfer to assess homeostatic proliferation, by flow-cytometric analysis of blood, spleen, and lymph-node cell suspensions prepared as described.

Log_2_-transformed output/input ratios of WT versus KO T cells were analysed separately for each tissue using one-sample *t*-tests against a theoretical mean of 0 (indicating no deviation from the input ratio). Because of the small sample size, Wilcoxon signed-rank tests were also performed; these gave qualitatively similar results. All analyses were performed in GraphPad Prism 10.

#### Edu proliferation assay

To assess cellular proliferation, mice were injected intraperitoneally with 250 µL of a 10 mg/mL EdU (5-ethynyl-2’-deoxyuridine) solution (resulting in a 2,5 mg EdU dose per mouse) 16 hours prior to sacrifice. Detection of incorporated EdU was performed according to the manufacturer’s protocol using the Click-iT™ EdU Alexa Fluor™ 647 (APC) Flow Cytometry Assay Kit (Thermo Fisher Scientific).

#### Seahorse

Naive cells (2,5x10^5^ cells/40µl/well) were spun onto 1 µg Poly-D-lysine-coated Seahorse 96 XF plates (at least 3 wells per sample) and filled 170 with XF assay buffer up to a total volume of 180 µl prior to pre-incubation for at least 45 min at 37 °C in the absence of CO_2_. Oxygen consumption rate (OCR) values were measured in basal conditions, but also after addition with the following respiratory inhibitors: 1 µM oligomycin, 1.5 µM FCCP, 0.1 µM rotenone + 1 µM antimycin A (all Sigma) using the Seahorse XF96 extracellular flux analyzer (Seahorse Bioscience, Agilent). Prior to each assay, inhibitors were made fresh from aliquoted frozen stocks and loaded into the injection ports of the XF-96 plate.

### Primary cell cultures

Naive splenic CD8^+^ T cells were isolated from 6–12 week old mice by FACS sorting (gated on live cells, CD8^+^ CD62L^+^ CD44^low^). Isolated T cells were either left unstimulated or were activated by plate-bound anti-mouse CD3 (experiment-specific concentrations are indicated in the respective figure legends, clone 145-2C11, Bioceros) and soluble CD28 (2 µg/ml, clone PV-1, Bioceros) in RPMI1640 supplemented with 10% FCS (Bodinco), 1.1 mg/ml b-mercapto-ethanol (Sigma), 56 µg/ml Gentamycin (Gibco) and 1% L-Glutamine (Gibco) for the indicated time points. Where indicated, cells were treated with the Rac inhibitor EHT1864 (MedChemExpress, #HY-16659) or the Cdc42 inhibitor ML141 (MedChemExpress, #HY-12755); specific concentrations are provided in the corresponding figure legends.

For cytoskeletal analyses, cells were stimulated with CCL19 (R&D Systems, #440-M3-025; concentration specified in figure legends) and immediately fixed and permeabilized using BD Cytofix/Cytoperm™ (BD Biosciences, #554714). F-actin was subsequently detected using Acti-stain Fluorescent Phalloidin 488 (Tebu-bio, #PHDG1-A).

For mitochondrial membrane potential rescue measurements, FACS-sorted naive T cells were cultured in phenol red-free RPMI supplemented with 2% FCS and 10 mM HEPES and allowed to rest for 30 min at 37 °C. Cells were subsequently treated with the NADPH oxidase inhibitor VAS2870 (MedChemExpress, #HY-12804, concentrations specified figure legends). After 90 min, TMRM (Thermo Fisher Scientific, #I34361) was added to assess mitochondrial membrane potential, and fluorescence was recorded 20 min later without washout.

### Analysis of protein expression by western blot

Immunoblotting: Following activation with anti-CD3/sCD28 for the indicated time points, cell extracts (± 500.000 purified naive CD8^+^ T cells/condition) were prepared in 30 µl E1A lysis buffer (1% NP40, 20 mM HEPES, pH 7.9, 250 mM NaCl, 1 mM EDTA) complemented with Complete-ULTRA (Roche) and PhosSTOP (Roche). Lysates were snap-frozen on dry ice and stored at −80 °C until further use for protein analysis by western blot. Prior to SDS–PAGE, samples were spun at 13,000g to remove cell debris and 5x loading dye was added. Following wet transfer to a nitrocellulose blotting membrane (GE Healthcare), proteins were visualized by chemiluminescence using Western lightning ECL plus (Perkin Elmer). Proteins were detected using MYC (sc-40; Santacruz Biotechnology; 1/500), β-TUBULIN (ab21058; Abcam; 1/5000), pERK1/2 (4370S, Cell Signaling, 1/1000) and ERK1/2 (4695S; Cell Signaling, 1/1000), pPKCθ (9377S, Cell Signaling, 1/1000) and PKCθ (13643S, Cell Signaling, 1/1000) antibodies.

#### Quantitative qPCR

Viral load determination was quantified using PVM *sh* gene mRNA levels on sap-frozen middle lobe samples. For this, frozen lung tissue was collected in a 2-ml microcentrifuge tube, and 1 ml of TriPure (Sigma-Aldrich) was added. Tissue was homogenized using a tissue homogenizer. To extract RNA, 200 μl of chloroform was added to the tubes containing the homogenized lung. After an incubation period of 5 min, tubes were centrifuged at 12,000g for 15 min. The upper transparent phase was collected in a ribonuclease (RNase)–free microcentrifuge tube and was mixed with 500 μl of isopropanol and 1 μl of glycogen for 10 min. The tubes were centrifuged at 12,000g for 5 min. The supernatant was discarded, and the pellet containing the purified RNA was washed in 75% ethanol (centrifugation at 7500g for 5 min). The pellet was resuspended in 20 μl of RNase-free water (Invitrogen). The tubes were placed for 10 min at 60 °C. RNA concentrations for each sample were determined using a NanoDrop instrument (Thermo Fisher Scientific). RNA (1 μg) was used to make cDNA using the SensiFAST cDNA Synthesis Kit (Bioline). The leftover RNA was frozen at −80 °C. The cDNA was diluted 10 times in water and frozen until further use. For real-time polymerase chain reaction (PCR), the following mastermix was used for each well of the PCR plate: 10 μl of SensiFAST SYBR No-ROX mix, 4.75 μl of water, 5 μl of cDNA, 0.125 μl of forward primer, and 0.125 μl of reverse primer (taken from a 100 μM stock). Primers were used as follows: PVM *sh* gene: forward ‘CGGGGGCGAATTTCTCCATA’, PVM *sh* gene: Reverse: ‘ATCAGCCAGCAGCTGAACAT’. Housekeeping gene RPL13a: Forward: ‘CCTGCTGCTCTCAAGGTTGTT’, Reverse: ‘TGGTTGTCACTGCCTGGTACTT’.

### Chemokine induced polarisation measurement

Naive CD8^+^ T cells were FACS-sorted and allowed to recover at 37 °C in RPMI supplemented with 0.1% BSA. Cells were transferred to a µ-Slide imaging chamber and imaged immediately upon stimulation with CCL19 (500 ng/mL) in the presence of fluorescent antibodies added in solution (anti-CD43 (553270, BD Biosciences) and anti-CD11a (101113, BioLegend)). Time-lapse imaging was performed on a Zeiss LSM 880 with Fast Airyscan. Polarisation was quantified by calculating the average intersect volume between CD43 and CD11a signals, normalised to the pre-stimulation timepoint (t = 0). Data represent the mean of 10 independent runs, with multiple cells imaged per run.

### Mitochondrial morphology analysis via TEM + scoring

FACS sorted naive CD8^+^ T cells were fixed in 4% PFA and 2.5% glutaraldehyde in 0.1 M NaCacodylate buffer, pH 7.2 and spun down at 1500 rpm. Low melting point agarose (1%) was used to keep the cells concentrated for further processing. The solidified agarose was cut in small pieces. Cells were washed three times in NaCacodylate buffer. After washing in buffer, they were post-fixed in 1% OsO4 with 1.5% K3Fe(CN)6 in 0.1 M NaCacodylate buffer at room temperature for 1 hour. After washing, cells were subsequently dehydrated through a graded ethanol series, including a bulk staining with 1% uranyl acetate at the 50% ethanol step followed by embedding in Spurr’s resin. Ultrathin sections of a gold interference color were cut using an ultramicrotome (Leica EM UC6), followed by a post-staining in a Leica EM AC20 for 40 min in uranyl acetate at 20 °C, and thereafter 10 min in lead stain at 20 °C. Sections were collected on Formvar-coated copper slot grids. Grids were viewed with a JEM 1400plus transmission electron microscope (JEOL, Tokyo, Japan) operating at 80 kV, and all images were taken at 5000X magnification.

Mitochondrial morphology was scored in a blinded manner on 207 WT and 334 KO mitochondria, derived from 56 WT and 72 KO images of randomly selected cells. Similarly to what has been previously described (67), mitochondria were classified as normal, minor defective or severe defective based on several criteria, including mitochondrial shape, intactness of the outer membrane, orientation/organisation/width/vagueness/absence of cristae, presence of mitochondrial remnants in autolysosomes and mitophagy. Image analysis was carried out using QuPath software.

### Proteomics

Following FACS sorting, cells were pelleted by centrifugation and snap-frozen, then stored at −80 °C until further processing for proteomic analysis.

#### Proteomics

LC-MS/MS runs of all 12 samples were searched together using the DiaNN (68) algorithm (version 2.2.0) with mainly default search settings, including a false discovery rate set at 1% on precursor and protein level. Protein groups identified with at least 2 peptides were kept. Spectra were searched against the Mus musculus protein sequences in the Uniprot database, containing 21,701 entries.

In all 12 samples: 50,955 precursors were identified. 47,559 peptides were identified. 45,392 of which were reliably quantified (= peptides quantified in at least 2 samples). 4,611 protein groups were reliably quantified (= protein groups quantified in at least 2 samples).

To compare proteins intensities between conditions, statistical testing for differences between group means was performed, using the package msqrob2 (69). Statistical significance for differential regulation was set at FDR < 0.05 and |log2FC| ≥ 2.

#### Phospho-proteomics

LC-MS/MS runs of all 12 samples were searched together using the MaxQuant (70) algorithm (version 2.6.7.0) with mainly default search settings, including a false discovery rate set at 1% on precursor and protein level. Phosphorylation was specified as variable modification. Protein groups identified with at least 2 peptides were kept. Spectra were searched against the *Mus musculus* protein sequences in the Uniprot database, containing 21,701 entries.

In all 12 samples: 23,405 precursor peptides (unique peptidoform-charge state combinations) were identified. 14,272 phosphopeptide precursors were identified (61%), corresponding to 2173 phosphopeptides. 504 of these were reliably quantified (= peptides with at least 3 valid intensity values in one of the experimental conditions) and belong to 320 proteins. These 504 different phosphopeptides represent 455 phosphosites. A single phosphosite can be quantified multiple times when it is identified alone as well as in combination with other sites on the same peptide, leading to redundancy in the list of quantified peptides.

To compare phosphosites intensities between conditions, statistical testing for differences between group means was performed, using the package msqrob2 (69). Missing intensity values were imputed by randomly sampling from a normal distribution centered around each sample’s noise level. Statistical significance for differential regulation was set at FDR < 0.05 and |log2FC| ≥ 2.

The mass spectrometry proteomics data have been deposited to the ProteomeXchange Consortium via the PRIDE partner repository with the dataset identifier PXD077760.

### Statistical analysis

Data are presented as mean ± SEM, as indicated in the figure legends. Normality was assessed using the Shapiro–Wilk test to determine the use of parametric or non-parametric statistical tests. For comparisons between two groups, either Welch’s *t*-test or the Mann-Whitney test was used, as appropriate. Comparisons among more than two groups were performed using one-way ANOVA with correction for multiple testing, or two-way ANOVA with *post-hoc* testing when two independent variables were involved. All statistical tests were two-sided, and statistical significance was defined as *P* < 0.05. Sample sizes were chosen according to standard guidelines, and the number of animals is indicated as *n*. All analyses were performed using GraphPad Prism 10 (GraphPad Software, La Jolla, CA). Detailed statistical parameters are provided in the figure legends. Investigators were not blinded to mouse group allocation.

## Data Availability

The mass spectrometry proteomics data have been deposited to the ProteomeXchange Consortium via the PRIDE partner repository with the dataset identifier PXD077760.

## Supplementary material

Supplementary Figure 1 Flow cytometric assessment of TCRVβ chain usage in splenic CD8^+^ T cells and thymic CD8^+^ single-positive (SP) cells (n=6).

Supplementary Figure 2 Overnight culture of FACS-sorted monoclonal naive CD8^+^ T cells stimulated with increasing concentrations of anti-CD3 in the presence of fixed anti-CD28 (2 µg/ml). Nur77-eGFP reporter activity was assessed by flow cytometry, with representative plots and quantification shown. The percentage of viable cells per condition is shown alongside (Two-way ANOVA statistical testing). Each data point represents an individual well. Data are representative of at least two independent experiments.

Supplementary Figure 3 Volcano plot of proteomic profiling of FACS-sorted naive CD8^+^ T cells, depicting differential protein abundance between experimental groups. Log_2_ fold change is plotted on the x-axis and −log_10_(*p*-value) on the y-axis. Significantly regulated proteins are highlighted in blue. (n=3)

Supplementary Figure 4 Actin polymerisation in naive CD8^+^ T cells following CCL19 (400 ng/mL) stimulation. Splenocytes were stimulated with CCL19 over time and gated on viable CD45^+^CD3^+^TCRβ^+^CD8^+^CD62L^+^CD44^low^ cells. F-actin content was assessed by phalloidin staining using flow cytometry, with ML141 (10 µM) or vehicle control. Quantification and representative flow cytometry plots of phalloidin intensity are shown.

## References

[1] Choi JO Seo Y Hwang SS . Guardians of silence: transcriptional networks in T cell quiescence. Exp Mol Med. (2025) 57:1663–72. doi: 10.1038/s12276-025-01516-y 40759740 PMC12411625

[2] Kawabe T Yi J Sprent J . Homeostasis of naive and memory T lymphocytes. Cold Spring Harbor Perspect Biol. (2021) 13:a037879. doi: 10.1101/cshperspect.a037879 33753403 PMC8411951

[3] Sprent J Surh CD . Normal T cell homeostasis: the conversion of naive cells into memory-phenotype cells. Nat Immunol. (2011) 12:478–84. doi: 10.1038/NI.2018 21739670 PMC3434123

[4] Mendoza A Fang V Chen C Serasinghe M Verma A Muller J . Lymphatic endothelial S1P promotes mitochondrial function and survival in naive T cells. Nature. (2017) 546:158–61. doi: 10.1038/nature22352 28538737 PMC5683179

[5] Schim van der Loeff I Hsu L-Y Saini M Weiss A Seddon B . Zap70 is essential for long-term survival of naive CD8 T cells. J Immunol. (2014) 193:2873–80. doi: 10.4049/jimmunol.1400858 25092893 PMC4167606

[6] Štefanoví I Dorfman JR Germain RN . Self-recognition promotes the foreign antigen sensitivity of naive T lymphocytes. Nature. (2002) 420:429–34. doi: 10.1038/nature01146 12459785

[7] Tan JT Dudl E LeRoy E Murray R Sprent J Weinberg KI . IL-7 is critical for homeostatic proliferation and survival of naïve T cells. PNAS. (2001) 98:8732–7. doi: 10.1073/PNAS.161126098 11447288 PMC37504

[8] Beemiller P Krummel MF . Regulation of T cell receptor signaling by the actin cytoskeleton and poroelastic cytoplasm. Immunol Rev. (2013) 256:10.1111/imr.12120. doi: 10.1111/IMR.12120 24117819 PMC3831008

[9] Blumenthal D Burkhardt JK . Multiple actin networks coordinate mechanotransduction at the immunological synapse. J Cell Biol. (2020) 219:e201911058. doi: 10.1083/JCB.201911058 31977034 PMC7041673

[10] Thauland TJ Hu KH Bruce MA Butte MJ . Cytoskeletal adaptivity regulates T cell receptor signaling. Sci Signaling. (2017) 10:eaah3737. doi: 10.1126/SCISIGNAL.AAH3737 28270556 PMC5854469

[11] Mosaddeghzadeh N Ahmadian MR . The RHO family GTPases: mechanisms of regulation and signaling. Cells. (2021) 10:1831. doi: 10.3390/CELLS10071831 34359999 PMC8305018

[12] Saoudi A Kassem S Dejean A Gaud G . Rho-GTPases as key regulators of T lymphocyte biology. Small GTPases. (2014) 5:e28208. doi: 10.4161/SGTP.28208 24825161 PMC4160340

[13] Gülow K Tümen D Heumann P Schmid S Kandulski A Müller M . Unraveling the role of reactive oxygen species in T lymphocyte signaling. Int J Mol Sci. (2024) 25:6114. doi: 10.3390/IJMS25116114 38892300 PMC11172744

[14] Hordijk PL . Regulation of NADPH oxidases: the role of Rac proteins. Circ Res. (2006) 98:453–62. doi: 10.1161/01.RES.0000204727.46710.5E 16514078

[15] Delpire E . The mammalian family of sterile 20p-like protein kinases. Pflügers Archiv - Eur J Physiol. (2009) 458:953–67. doi: 10.1007/s00424-009-0674-y 19399514

[16] Fang CY Lai TC Hsiao M Chang YC . The diverse roles of TAO kinases in health and diseases. Int J Mol Sci. (2020) 21:7463. doi: 10.3390/IJMS21207463 33050415 PMC7589832

[17] Hammad H Vanderkerken M Pouliot P Deswarte K Toussaint W Vergote K . Transitional B cells commit to marginal zone B cell fate by Taok3-mediated surface expression of ADAM10. Nat Immunol. (2017) 18:313–20. doi: 10.1038/ni.3657 28068307

[18] Maes B Smole U Vanderkerken M Deswarte K Van Moorleghem J Vergote K . The STE20 kinase TAOK3 controls the development of house dust mite–induced asthma in mice. J Allergy Clin Immunol. (2022) 149:1413–1427.e2. doi: 10.1016/j.jaci.2021.08.020 34506849

[19] Vanderkerken M Maes B Vandersarren L Toussaint W Deswarte K Vanheerswynghels M . TAO-kinase 3 governs the terminal differentiation of NOTCH2-dependent splenic conventional dendritic cells. PNAS. (2020) 117:31331–42. doi: 10.1073/pnas.2009847117 33214146 PMC7733863

[20] Ormonde JVS Li Z Stegen C Madrenas J . TAOK3 regulates canonical TCR signaling by preventing early SHP-1–mediated inactivation of LCK. J Immunol. (2018) 201:3431–42. doi: 10.4049/jimmunol.1800284 30373850

[21] Poirier A Ormonde JVS Aubry I Abidin BM Feng CH Martinez-Cordova Z . The induction of SHP-1 degradation by TAOK3 ensures the responsiveness of T cells to TCR stimulation. Sci Signaling. (2024) 17(817). doi: 10.1126/scisignal.adg4422 38166031

[22] Yoneda T Imaizumi K Oono K Yui D Gomi F Katayama T . Activation of caspase-12, an endoplastic reticulum (ER) resident caspase, through tumor necrosis factor receptor-associated factor 2-dependent mechanism in response to the ER stress. J Biol Chem. (2001) 276:13935–40. doi: 10.1074/JBC.M010677200 11278723

[23] Maes B Fayazpour F Catrysse L Lornet G Van De Velde E De Wolf C . STE20 kinase TAOK3 regulates type 2 immunity and metabolism in obesity. J Exp Med. (2023) 220(9). doi: 10.1084/jem.20210788 37347461 PMC10287548

[24] Bosteels C Neyt K Vanheerswynghels M van Helden MJ Sichien D Debeuf N . Inflammatory type 2 cDCs acquire features of cDC1s and macrophages to orchestrate immunity to respiratory virus infection. Immunity. (2020) 52:1039–56. doi: 10.1016/j.immuni.2020.04.005 32392463 PMC7207120

[25] Min B . Spontaneous T cell proliferation: a physiologic process to create and maintain homeostatic balance and diversity of the immune system. Front Immunol. (2018) 9:547/BIBTEX:353709. doi: 10.3389/fimmu.2018.00547 29616038 PMC5868360

[26] Goldrath AW Bogatzki LY Bevan MJ . Naive T cells transiently acquire a memory-like phenotype during homeostasis-driven proliferation. J Exp Med. (2000) 192:557–64. doi: 10.1084/JEM.192.4.557 10952725 PMC2193243

[27] Moran AE Holzapfel KL Xing Y Cunningham NR Maltzman JS Punt J . T cell receptor signal strength in Treg and iNKT cell development demonstrated by a novel fluorescent reporter mouse. J Exp Med. (2011) 208:1279–89. doi: 10.1084/jem.20110308 21606508 PMC3173240

[28] Zikherman J Parameswaran R Weiss A . Endogenous antigen tunes the responsiveness of naive B cells but not T cells. Nature. (2012) 489:160–4. doi: 10.1038/nature11311 22902503 PMC3438375

[29] Štefanová I Hemmer B Vergelli M Martin R Biddison WE Germain RN . TCR ligand discrimination is enforced by competing ERK positive and SHP-1 negative feedback pathways. Nat Immunol. (2003) 4:248–54. doi: 10.1038/ni895 12577055

[30] Barouch-Bentov R Lemmens EE Hu J Janssen EM Droin NM Song J . Protein kinase C-θ is an early survival factor required for differentiation of effector CD8+ T cells. J Immunol. (2005) 175:5126–34. doi: 10.4049/jimmunol.175.8.5126 16210616

[31] Mandl JN Monteiro JP Vrisekoop N Germain RN . T cell-positive selection uses self-ligand binding strength to optimize repertoire recognition of foreign antigens. Immunity. (2013) 38:263–74. doi: 10.1016/j.immuni.2012.09.011 23290521 PMC3785078

[32] Bem RA Domachowske JB Rosenberg HF . Animal models of human respiratory syncytial virus disease. (2011) 301:148–56. doi: 10.1152/ajplung.00065.2011 PMC3154630 21571908

[33] Frey S Krempl CD Schmitt-Gräff A Ehl S . Role of T cells in virus control and disease after infection with pneumonia virus of mice. J Virol. (2008) 82:11619–27. doi: 10.1128/jvi.00375-08 18815308 PMC2583671

[34] Vandersarren L Bosteels C Vanheerswynghels M Moon JJ Easton AJ Van Isterdael G . Epitope mapping and kinetics of CD4 T cell immunity to pneumonia virus of mice in the C57BL/6 strain. Sci Rep. (2017) 7:2–11. doi: 10.1038/s41598-017-03042-y 28615708 PMC5471230

[35] Walsh KB Sidney J Welch M Fremgen DM Sette A Oldstone MBA . CD8 + T-cell epitope mapping for pneumonia virus of mice in H-2 b mice. J Virol. (2013) 87:9949–52. doi: 10.1128/jvi.00339-13 23824814 PMC3754129

[36] Grieve AG Moss SE Hayes MJ . Annexin A2 at the interface of actin and membrane dynamics: a focus on its roles in endocytosis and cell polarization. Int J Cell Biol. (2012) 2012:852430. doi: 10.1155/2012/852430 22505935 PMC3296266

[37] Saponaro C Gammaldi N Cavallo V Ramírez-Morales MA Zito FA Sonnessa M . Insight into the regulation of NDRG1 expression. Int J Mol Sci. (2025) 26:3582. doi: 10.3390/ijms26083582 40332138 PMC12027247

[38] Bagci H Sriskandarajah N Robert A Boulais J Elkholi IE Tran V . Mapping the proximity interaction network of the Rho-family GTPases reveals signalling pathways and regulatory mechanisms. Nat Cell Biol. (2019) 22:120–34. doi: 10.1038/s41556-019-0438-7 31871319

[39] Capece T Abid A Kim M . Regulation of integrin LFA-1 (CD11a/CD18) during T cell migration and activation (CAM1P.227). J Immunol. (2014) 192:47.3–3. doi: 10.4049/jimmunol.192.supp.47.3

[40] Del Pozo MA Cabañas C Montoya MC Ager A Sánchez-Mateos P Sánchez-Madrid F . ICAMs redistributed by chemokines to cellular uropods as a mechanism for recruitment of T lymphocytes. J Cell Biol. (1997) 137:493–508. doi: 10.1083/jcb.137.2.493 9128258 PMC2139764

[41] Bailly C Degand C Laine W Sauzeau V Kluza J . Implication of Rac1 GTPase in molecular and cellular mitochondrial functions. Life Sci. (2024) 342:122510. doi: 10.1016/j.lfs.2024.122510 38387701

[42] Ferro E Goitre L Retta SF Trabalzini L . The interplay between ROS and Ras GTPases: physiological and pathological implications. J Signal Transduction. (2012) 2012:365769. doi: 10.1155/2012/365769 22175014 PMC3235814

[43] Brand MD Nicholls DG . Assessing mitochondrial dysfunction in cells. Biochem J. (2011) 435:297–312. doi: 10.1042/BJ20110162 21726199 PMC3076726

[44] van der Windt GJW Pearce EL . Metabolic switching and fuel choice during T-cell differentiation and memory development. Immunol Rev. (2012) 249:27–42. doi: 10.1111/j.1600-065X.2012.01150.x 22889213 PMC3645891

[45] Kroemer G Mariño G Levine B . Autophagy and the integrated stress response. Mol Cell. (2010) 40:280–93. doi: 10.1016/j.molcel.2010.09.023 20965422 PMC3127250

[46] Cotta-de-Almeida V Westerberg L Maillard MH Onaldi D Wachtel H Meelu P . Wiskott Aldrich syndrome protein (WASP) and N-WASP are critical for T cell development. PNAS. (2007) 104:15424–9. doi: 10.1073/PNAS.0706881104 17878299 PMC2000553

[47] Fujikawa K Miletic AV Alt FW Faccio R Brown T Hoog J . Vav1/2/3-null mice define an essential role for Vav family proteins in lymphocyte development and activation but a differential requirement in MAPK signaling in T and B cells. J Exp Med. (2003) 198:1595–608. doi: 10.1084/JEM.20030874 14623913 PMC2194126

[48] Randall KL Chan SSY Ma CS Fung I Mei Y Yabas M . DOCK8 deficiency impairs CD8 T cell survival and function in humans and mice. J Exp Med. (2011) 208:2305. doi: 10.1084/JEM.20110345 22006977 PMC3201196

[49] Roy E Togbe D Holdorf AD Trubetskoy D Nabti S Küblbeck G . Nck adaptors are positive regulators of the size and sensitivity of the T-cell repertoire. PNAS. (2010) 107:15529–34. doi: 10.1073/pnas.1009743107 20709959 PMC2932578

[50] Janssen E Tohme M Hedayat M Leick M Kumari S Ramesh N . A DOCK8-WIP-WASp complex links T cell receptors to the actin cytoskeleton. J Clin Invest. (2016) 126:3837–51. doi: 10.1172/JCI85774 27599296 PMC5096816

[51] Tao X Wu Y Guo F Lv L Zhai X Shang D . Targeted inhibitors of S100A9 alleviate chronic pancreatitis by inhibiting M2 macrophage polarization via the TAOK3-JNK signaling pathway. Front Immunol. (2025) 16:1526813/BIBTEX. doi: 10.3389/fimmu.2025.1526813 40207228 PMC11979270

[52] Shen C Cerf A Postat J Bhagrath A Merino M Mingarelli A . A Dock8-dependent mechanosensitive central actin pool maintains T cell shape and protects the nucleus during migration. Sci Immunol. (2025) 10(109). doi: 10.1126/sciimmunol.adt9239 40570086

[53] Krishnaswamy JK Gowthaman U Zhang B Mattsson J Szeponik L Liu D . Migratory CD11b+ conventional dendritic cells induce T follicular helper cell–dependent antibody responses. Sci Immunol. (2017) 2:eaam9169. doi: 10.1126/sciimmunol.aam9169 29196450 PMC7847246

[54] Randall KL Lambe T Johnson A Treanor B Kucharska E Domaschenz H . Dock8 mutations cripple B cell immunological synapses, germinal centers and long-lived antibody production. Nat Immunol. (2009) 10:1283–91. doi: 10.1038/ni.1820 19898472 PMC3437189

[55] van der Windt GJW Everts B Chang CH Curtis JD Freitas TC Amiel E . Mitochondrial respiratory capacity is a critical regulator of CD8+ T cell memory development. Immunity. (2012) 36:68–78. doi: 10.1016/J.IMMUNI.2011.12.007 22206904 PMC3269311

[56] Myers DR Zikherman J Roose JP . Tonic signals: why do lymphocytes bother? Trends Immunol. (2017) 38:844–57. doi: 10.1016/j.it.2017.06.010 28754596 PMC5669999

[57] Tanchot C Lemonnier FA Pérarnau B Freitas AA Rocha B . Differential requirements for survival and proliferation of CD8 naïve or memory T cells. Science. (1997) 276:2057–62. doi: 10.1126/science.276.5321.2057 9197272

[58] Garg R Koo CY Infante E Giacomini C Ridley AJ Morris JDH . Rnd3 interacts with TAO kinases and contributes to mitotic cell rounding and spindle positioning. J Cell Sci. (2020) 133(6). doi: 10.1242/jcs.235895 32041905

[59] Johne C Matenia D Li XY Timm T Balusamy K Mandelkow EM . Spred1 and TESK1—two new interaction partners of the kinase MARKK/TAO1 that link the microtubule and actin cytoskeleton. (2008) 19:1391–403. doi: 10.1091/MBC.E07-07-0730 PMC2291396 18216281

[60] Mitsopoulos C Zihni C Garg R Ridley AJ Morris JDH . The prostate-derived sterile 20-like kinase (PSK) regulates microtubule organization and stability. J Biol Chem. (2003) 278:18085–91. doi: 10.1074/jbc.M213064200 12639963

[61] Nourbakhsh K Ferreccio AA Bernard MJ Yadav S . TAOK2 is an ER-localized kinase that catalyzes the dynamic tethering of ER to microtubules. Dev Cell. (2021) 56:3321–3333.e5. doi: 10.1016/J.DEVCEL.2021.11.015 34879262 PMC8699727

[62] Richter M Murtaza N Scharrenberg R White SH Johanns O Walker S . Altered TAOK2 activity causes autism-related neurodevelopmental and cognitive abnormalities through RhoA signaling. Mol Psychiatry. (2018) 24:1329–50. doi: 10.1038/s41380-018-0025-5 29467497 PMC6756231

[63] Timm T Li XY Biernat J Jiao J Mandelkow E Vandekerckhove J . MARKK, a Ste20‐like kinase, activates the polarity‐inducing kinase MARK/PAR‐1. EMBO J. (2003) 22:5090–101. doi: 10.1093/EMBOJ/CDG447 14517247 PMC204455

[64] Lai TC Fang CY Jan YH Hsieh HL Yang YF Liu CY . Kinase shRNA screening reveals that TAOK3 enhances microtubule-targeted drug resistance of breast cancer cells via the NF-κB signaling pathway. Cell Commun Signaling. (2020) 18:164. doi: 10.1186/S12964-020-00600-2 33087151 PMC7579951

[65] Quadros RM Miura H Harms DW Akatsuka H Sato T Aida T . Easi-CRISPR: a robust method for one-step generation of mice carrying conditional and insertion alleles using long ssDNA donors and CRISPR ribonucleoproteins. Genome Biol. (2017) 18, 92. doi: 10.1186/s13059-017-1220-4 28511701 PMC5434640

[66] Cook PM Eglin RP Easton AJ . Pathogenesis of pneumovirus infections in mice: Detection of pneumonia virus of mice and human respiratory syncytial virus mRNA in lungs of infected mice by in situ hybridization. J Gen Virol. (1998) 79:2411–7. doi: 10.1099/0022-1317-79-10-2411 9780046

[67] Xiong ZM Choi JY Wang K Zhang H Tariq Z Wu D . Methylene blue alleviates nuclear and mitochondrial abnormalities in progeria. Aging Cell. (2016) 15:279–90. doi: 10.1111/acel.12434 26663466 PMC4783354

[68] Demichev V Messner CB Vernardis SI Lilley KS Ralser M . DIA-NN: neural networks and interference correction enable deep proteome coverage in high throughput. Nat Methods. (2019) 17:41–4. doi: 10.1038/s41592-019-0638-x 31768060 PMC6949130

[69] Sticker A Goeminne L Martens L Clement L . Robust summarization and inference in proteome-wide label-free quantification. Mol Cell Proteomics. (2020) 19:1209–19. doi: 10.1074/mcp.RA119.001624 32321741 PMC7338080

[70] Cox J Mann M . MaxQuant enables high peptide identification rates, individualized p.p.b.-range mass accuracies and proteome-wide protein quantification. Nat Biotechnol. (2008) 26:1367–72. doi: 10.1038/nbt.1511 19029910

