# Supplementary figures and images for: The Ste20 kinase TAOK3 restrains Rac-driven cytoskeletal-mitochondrial coupling to preserve naive CD8+ T cell homeostasis and activation
Source: Front Immunol. 2026 Jun 15;17:1838764. doi: 10.3389/fimmu.2026.1838764 (PMC13311001; doi:10.3389/fimmu.2026.1838764)

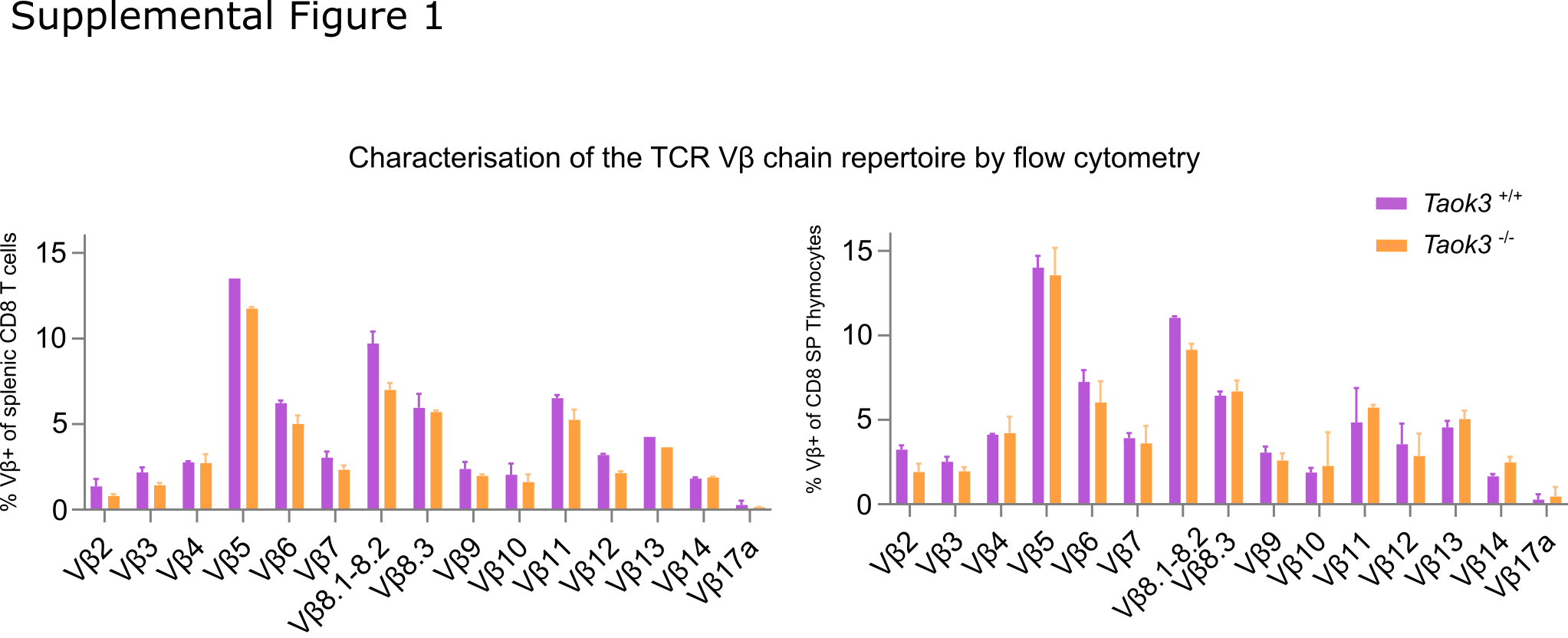

Supplement: Supplementary Figure 1 — Flow cytometric assessment of TCRVβ chain usage in splenic CD8+ T cells and thymic CD8+ single-positive (SP) cells (n=6). [file Image1.tiff]

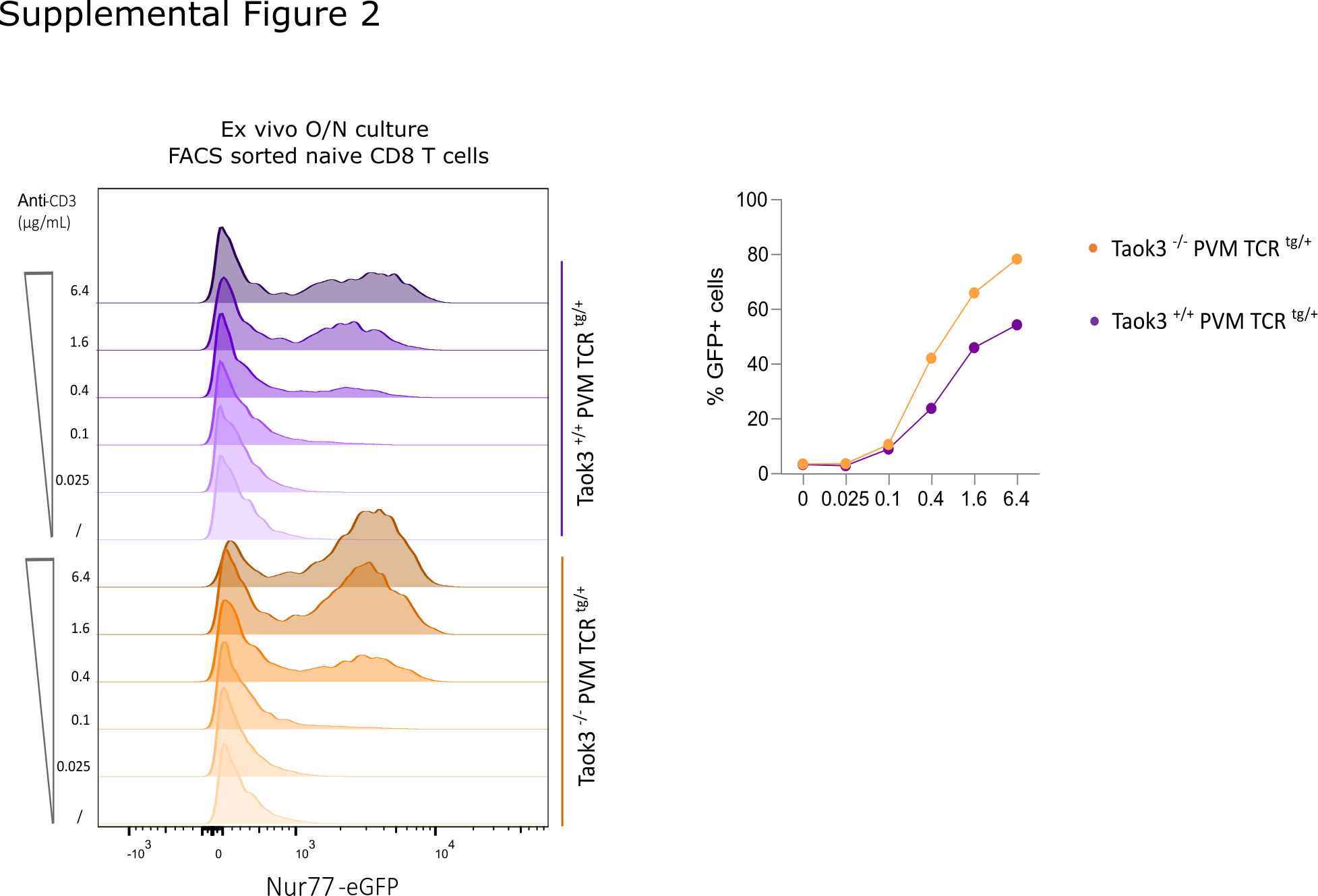

Supplement: Supplementary Figure 2 — Overnight culture of FACS-sorted monoclonal naive CD8+ T cells stimulated with increasing concentrations of anti-CD3 in the presence of fixed anti-CD28 (2 µg/ml). Nur77-eGFP reporter activity was assessed by flow cytometry, with representative plots and quantification shown. The percentage of viable cells per condition is shown alongside (Two-way ANOVA statistical testing). Each data point represents an individual well. Data are representative of at least two independent experiments. [file Image2.tiff]

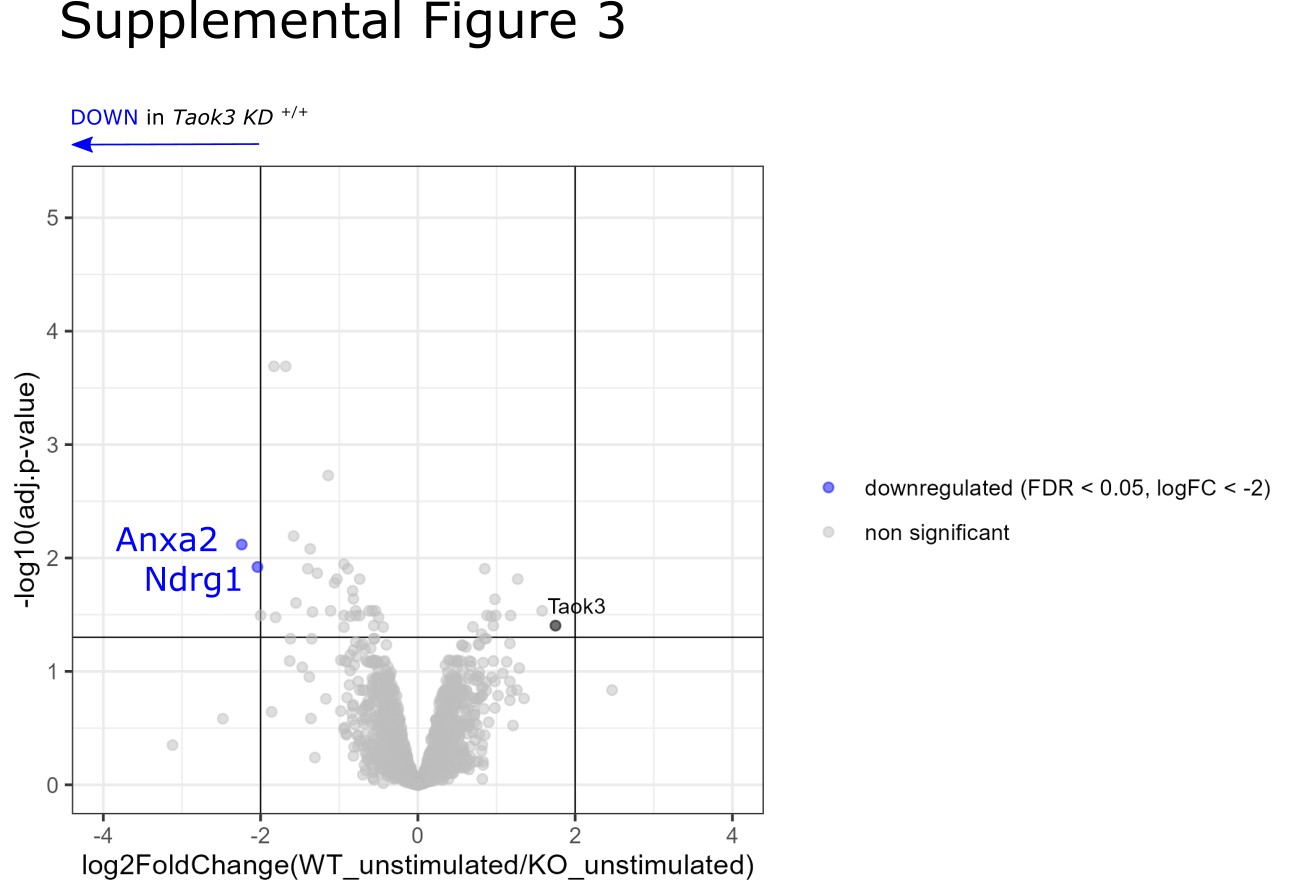

Supplement: Supplementary Figure 3 — Volcano plot of proteomic profiling of FACS-sorted naive CD8+ T cells, depicting differential protein abundance between experimental groups. Log2 fold change is plotted on the x-axis and −log10(p-value) on the y-axis. Significantly regulated proteins are highlighted in blue. (n=3) [file Image3.jpeg]

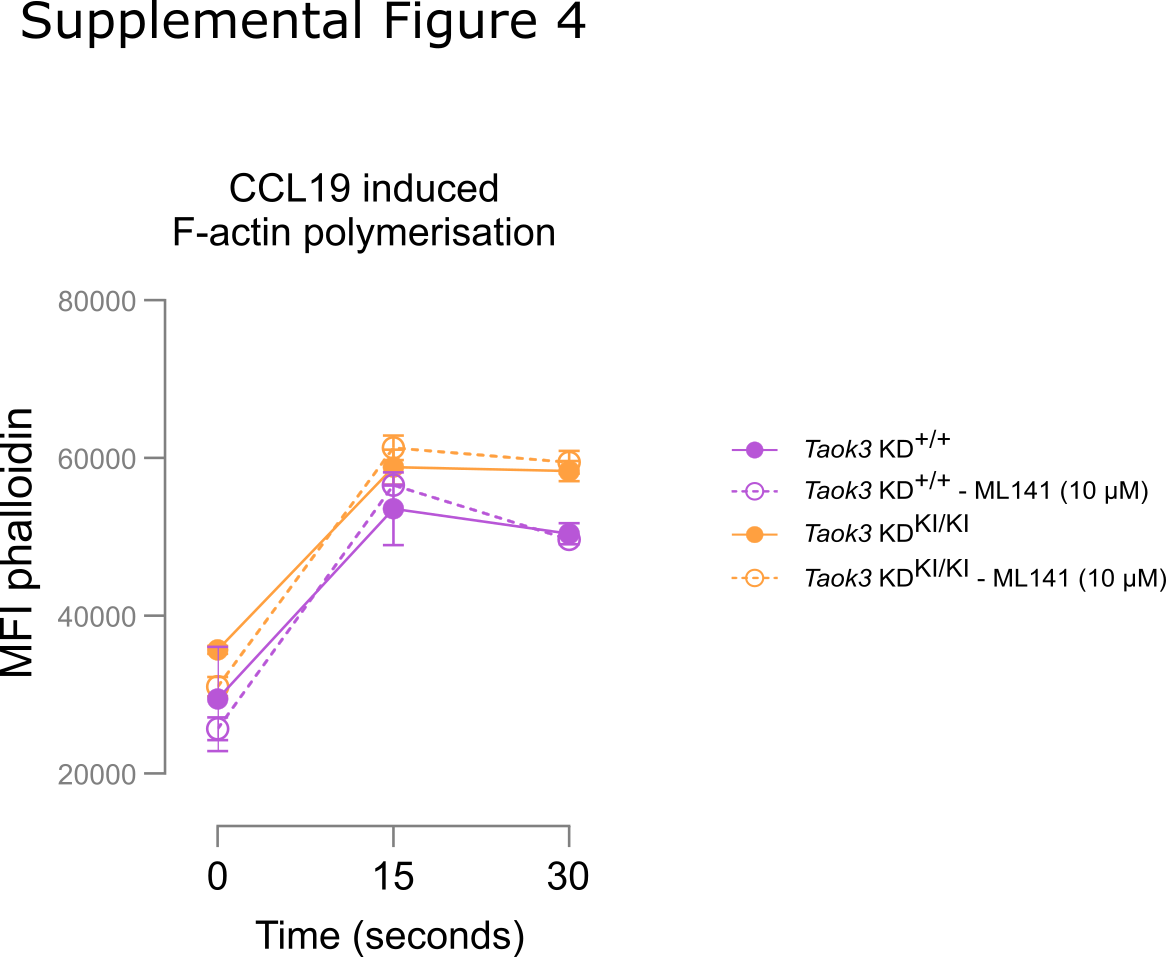

Supplement: Supplementary Figure 4 — Actin polymerisation in naive CD8+ T cells following CCL19 (400 ng/mL) stimulation. Splenocytes were stimulated with CCL19 over time and gated on viable CD45+CD3+TCRβ+CD8+CD62L+CD44low cells. F-actin content was assessed by phalloidin staining using flow cytometry, with ML141 (10 µM) or vehicle control. Quantification and representative flow cytometry plots of phalloidin intensity are shown. [file Image4.tiff]
